# Peroxisomes in Different Skeletal Cell Types during Intramembranous and Endochondral Ossification and Their Regulation during Osteoblast Differentiation by Distinct Peroxisome Proliferator-Activated Receptors

**DOI:** 10.1371/journal.pone.0143439

**Published:** 2015-12-02

**Authors:** Guofeng Qian, Wei Fan, Barbara Ahlemeyer, Srikanth Karnati, Eveline Baumgart-Vogt

**Affiliations:** Institute for Anatomy and Cell Biology, Medical Cell Biology, Justus-Liebig-University, Aulweg 123, 35385 Giessen, Germany; Kyungpook National University School of Medicine, REPUBLIC OF KOREA

## Abstract

Ossification defects leading to craniofacial dysmorphism or rhizomelia are typical phenotypes in patients and corresponding knockout mouse models with distinct peroxisomal disorders. Despite these obvious skeletal pathologies, to date no careful analysis exists on the distribution and function of peroxisomes in skeletal tissues and their alterations during ossification. Therefore, we analyzed the peroxisomal compartment in different cell types of mouse cartilage and bone as well as in primary cultures of calvarial osteoblasts. The peroxisome number and metabolism strongly increased in chondrocytes during endochondral ossification from the reserve to the hypertrophic zone, whereas in bone, metabolically active osteoblasts contained a higher numerical abundance of this organelle than osteocytes. The high abundance of peroxisomes in these skeletal cell types is reflected by high levels of *Pex11*β gene expression. During culture, calvarial pre-osteoblasts differentiated into secretory osteoblasts accompanied by peroxisome proliferation and increased levels of peroxisomal genes and proteins. Since many peroxisomal genes contain a PPAR-responsive element, we analyzed the gene expression of PPARɑ/ß/ɣ in calvarial osteoblasts and MC3T3-E1 cells, revealing higher levels for PPARß than for PPARɑ and PPARɣ. Treatment with different PPAR agonists and antagonists not only changed the peroxisomal compartment and associated gene expression, but also induced complex alterations of the gene expression patterns of the other PPAR family members. Studies in M3CT3-E1 cells showed that the PPARß agonist GW0742 activated the PPRE-mediated luciferase expression and up-regulated peroxisomal gene transcription (*Pex11*, *Pex13*, *Pex14*, *Acox1* and *Cat*), whereas the PPARß antagonist GSK0660 led to repression of the PPRE and a decrease of the corresponding mRNA levels. In the same way, treatment of calvarial osteoblasts with GW0742 increased in peroxisome number and related gene expression and accelerated osteoblast differentiation. Taken together, our results suggest that PPARß regulates the numerical abundance and metabolic function of peroxisomes via *Pex11ß* in parallel to osteoblast differentiation.

## Introduction

Peroxisomes are ubiquitous organelles in eukaryotic cells that play a central role in lipid and reactive oxygen species metabolism (reviewed by [1]). Peroxisomes arise “de novo” and by division of pre-existing organelles. Peroxisome biogenesis is mediated by more than 32 PEX genes and their corresponding gene products, the peroxins. Peroxins are responsible for the synthesis of the peroxisomal membrane (e.g. PEX3, PEX19), the matrix import (e.g. PEX2, PEX5, PEX7, PEX13 and PEX14) and proliferation of peroxisomes (e.g. PEX11 family) [2]. The importance of these organelles for the development of the skeleton is best demonstrated in patients suffering from peroxisomal biogenesis disorders (PBDs) leading to a complete disruption of peroxisomal metabolic function. Children with Zellweger syndrome, the most severe form of PBDs, exhibit a general growth retardation, a craniofacial dysmorphism including a high forehead, a broad nasal bridge, hypertelorism, shallow orbital ridges, a high arched palate, large fontanelles, and a flat occiput [3]. In addition, in humans suffering from rhizomelic chondrodysplasia punctata type 1, caused by a defective *PEX7* gene [4,5], stippled foci of calcification within hyaline cartilage, dwarfism due to symmetrical shortening of proximal long bones (rhizomelia) and coronal clefting of the vertebrae were observed [6,7]. Most corresponding knockout mouse models (e.g. for *Pex5* [8]; for *Pex11ß* [9]; for *Pex13* [10]) showed a general growth retardation. Moreover, in *Pex2* [11] and *Pex7* knockout mice [12], skull defects were described indicating abnormal intramembranous (*Pex2;* calvaria) and endochondral (*Pex7*, basis sphenoid and inner ear ossicles) ossification. Moreover, skeletal abnormalities were found in the distal bone elements of the limbs as well as in vertebrae of newborn Pex7 knockout mice [12]. Similarly, in a hypomorphic *Pex7* ^neo/neo^ mouse, exhibiting less *Pex7* gene transcripts, a delayed endochondral ossification was noted already at postnatal day 1 and the adult animals (10 weeks of age) were petite [13].

Despite the severe ossification defects observed in patients and knockout mice with PBDs, no detailed study on the normal distribution, abundance and enzyme composition of peroxisomes in the skeleton is yet available. Moreover, the regulation of the peroxisomal compartment and corresponding gene transcription during osteoblast differentiation and maturation is unknown. Interestingly, PPARɑ, known to bind lipid ligands and to activate the transcription of peroxisomal genes [14,15], but also PPARß and PPARɣ were shown to modulate osteoblast differentiation (reviewed by [16]). In addition, many PPAR lipid ligands are degraded by peroxisomal β-oxidation suggesting a possible peroxisome-PPAR loop for the control of PPAR ligand homeostasis (reviewed by [17]). Indeed, PPARɑ is present in osteoblasts and its activation by bezafibrate stimulated osteoblast differentiation [18], even though PPARɑ knockout mice did not show an obvious bone phenotype [19]. PPARß was recently shown to serve as a key regulator of bone turnover and of the crosstalk between osteoclasts and osteoblasts through Wnt- and β-catenin dependent signaling [20], whereas, PPARɣ activation negatively regulates osteoblast differentiation and transforms mesenchymal stem cells into the adipocyte lineage [21].

In this study, we characterized the distribution, numerical abundance and enzyme composition of peroxisomes in different cell types of the mouse skeleton during endochondral and intramembranous ossification, as well as in differentiating primary osteoblast cultures from the mouse calvaria. Furthermore, we analyzed the effects of different PPAR agonists and antagonists on peroxisome proliferation and metabolic function as well as on the expression of all three PPAR genes. We show that mainly PPARß activation is responsible for PPRE-mediated maturation of the peroxisomal compartment and for the differentiation and maturation of osteoblasts.

## Materials and Methods

### 1. Materials

Collagenase II and fetal calf serum (FCS) were purchased from PAA (Cölbe, Germany). ɑ-Minimum Essential Medium (ɑ-MEM), DNase I, oligo (dT) 12–18 primers, superscript II reverse transcriptase, TOTO-3-iodide were from Invitrogen (Karlsruhe, Germany), and glycerol 2-phosphate disodium salt, L-ascorbic acid, Alizarin Red S, Tween 20, Hoechst 33342, NP-40, ciprofibrate, troglitazone, GW9662, β-mercaptoethanol, poly-L-lysine, proteinase K, Denhardt’s solution, nitroblue tetrazolium salt, 5-bromo-4-chloro-3-indolyl phosphate, levamisole and bovine serum albumin (BSA) were from Sigma-Aldrich (Deisenhofen, Germany). GW6471, GW0742 and GSK0660 were purchased from TOCRIS distributed by R&D Systems (Wiesbaden, Germany). The Dual-Luciferase® Reporter Assay System (Cat. E1910) was bought from Promega (Mannheim, Germany). Alkaline phosphatase-labeled anti-digoxigenin Fab fragments and the respective blocking medium were derived from Boehringer Mannheim (Mannheim, Germany). The protease inhibitor mix M was from Serva (Heidelberg, Germany) and Immun-Star™ AP substrate and SYBR® Gold from Bio-Rad Laboratories (München, Germany). All primary and secondary antibodies used in this study were listed in Tables 1 and 2. The RNeasy Mini Kit and the PPAR Reporter Kit (Cat. CCS-3026L) were obtained from Qiagen (Hilden, Germany). The 5 PRIME TaqDNA polymerase, dNTPs and the 5 PRIME Master Mix were all from 5 PRIME (Hamburg, Germany). Maxima SYBR Green qPCR Master Mix (Cat. K0243) was purchased from Thermo Scientific (Dreieich, Germany). Primers for semiquantitative and quantitative reverse transcriptase (RT)-PCR were synthesized by Eurofins (Ebersberg, Germany); sequences, number of cycles and efficiency coefficients were given in Table 2. Mouse genes and proteins were named according to the official NIH nomenclature throughout the manuscript.

**Table 1:** List of primary antibodies used in this study.

Target antigen	Host	Source/Catalog number	Dilution IF	Dilution WB
ABCD3, rat	Rb	Invitrogen, Karlsruhe, Germany; Cat. 71–8300	1:1,000	
ABCD3, mouse	Rb	Gift from Alfred Völkl, Ruprecht-Karls-University, Heidelberg, Germany		1:100
ALP, human	Sh	Acris Antibodies GmbH, Hiddenhausen, Germany; Cat. BP237	1:1,000	
Catalase, mouse	Rb	Gift from Denis Crane, Griffith University, Brisbane, Australia	1:2,000	1:10,000
Cathepsin K, human	Gt	Santa Cruz Biotechnology, Heidelberg, Germany; Cat. Sc-6506	1:400	
Ki67, mouse	Rt	Dako Cytomation, Denmark; Cat. M7249	1:6,000	
OPN, mouse	Ms	Developmental Studies Hybridoma Bank (DSHB), University of Iowa, Iowa City, US; Cat. MPIIIB101	1:2,000	1:50,000
Osteocalcin, human	Ms	R&D Systems, Wiesbaden, Germany; Cat. MAB1419	1:100	
PEX5, mouse	Ms	BD Transduction Laboratories, USA; Cat. No 611594		1:200
PEX13, mouse	Rb	Gift from Denis Crane, Griffith University, Brisbane, Australia	1:2,000	1:6,000
PEX14, mouse	Rb	Gift from Denis Crane, Griffith University, Brisbane, Australia	1:4,000	1:20,000
SKL, mouse	Rb	Invitrogen, Karlsruhe, Germany; Cat. 71–8400	1:400	
SOD2, rat	Rb	RDI Research Diagnostics, NJ, US; Cat. RDI-RTSODMabR		1:6,000
Thiolase, mouse	Ms	Gift from Paul van Veldhoven, Catholic University of Leuven, Leuven, Belgium		1:1,000
ɑ-Tubulin, mouse	Ms	Sigma, Steinheim, Germany; Cat. T5168		1:5,000
UQCRC2, human	Ms	Invitrogen, Karlsruhe, Germany; Cat. A11143		1:1,000

**Table 2:** List of secondary antibodies used in this study.

Target antigen	Host	Source/Catalog number	Dilution IF	Dilution WB
Anti-Rabbit IgG Alexa Fluor 488	Do	Invitrogen/Molecular Probes, Darmstadt, Germany; Cat. A21206	1:600	
Anti-Mouse IgG Texas Red	Hs	Vector Laboratories, Inc, Burlingame, USA, Cat. TI-2000	1:200	
Anti-Sheep IgG Rhodamine Red	Do	Dianova, Hamburg, Germany; Cat. 713-295-147	1:600	
Anti-Rat IgG Alexa Fluor 594	Gt	Invitrogen/Molecular Probes, Darmstadt, Germany; Cat. A11007	1:600	
Anti-Goat IgG Alexa Fluor 594	Ch	Invitrogen/Molecular Probes, Darmstadt, Germany; Cat. A21468	1:500	
Anti-Mouse IgG ALP	Gt	Sigma-Aldrich, Deisenhofen, Germany; Cat. A3562		1:20,000
Anti-Rabbit IgG ALP	Gt	Sigma-Aldrich, Deisenhofen, Germany; Cat. A7872		1:20,000

### 2. Animals

Five C57Bl/6J mice at the age of 40 days and thirteen pregnant mice (to obtain newborn pups) were purchased from Charles River Laboratories (Sulzfeld, Germany). All animals had free access to food and water and were kept under standardized environmental conditions (12 h light/dark cycle, 23°C ± 1°C and 55% ± 1% relative humidity). This study was carried out in strict accordance with the recommendation of the national guide for the care and use of laboratory animals (Deutsches Tierschutzgesetz). The protocol was approved by the German Government Commission of Animal Care, (Regierungspräsidium Gießen; Permit Number V54-19 c 20/15 c GI 20/23). All surgery was performed under ketamine and xylazine anaesthesia and all efforts were made to minimize suffering.

### 3. Perfusion Fixation of Adult and Newborn Mice and Processing of Mouse Tissues for Paraffin Embedding and Sectioning

Five wild-type C57Bl/6J mice at the age of 40 days and 13 newborn mouse pups were anesthetized and perfused through the left ventricle of heart with freshly prepared 4% depolymerized paraformaldehyde in 0.01 M phosphate-buffered saline (PBS, pH 7.4). Mouse femora, calvaria and vertebrae from the 40 days-old mice were dissected out, immersion-fixed overnight in the same fixative and then decalcified with 10% EDTA at 4°C for 7 days. Newborn mice were additionally immersion-fixated for 24 h in the same fixative and then cut sagittally in two halves. Thereafter, the specimen were embedded into paraffin using a Leica TP 1020 automated vacuum infiltration tissue processor using the following steps, 90 min each: 70%, 80%, 90%, 3 x 100% ethanol; 2 h each: 2 x xylene, 2 x paraffin. Paraffin blocks were cut into sections of 3–4 μm thickness and mounted on Superfrost® Plus slides (Labor- und Medizintechnik, Emmendingen, Germany). For an overview on bone architecture, paraffin sections of the calvaria and mandible (P0.5 mice) were counterstained with hematoxylin and eosin. The basophilic structures (e.g. nuclei) appear in violet and the acidic ones (e.g. bone matrix, osteoclasts and erythrocytes) in red colors.

### 4. Indirect Immunofluorescence on Paraffin Sections for Localization of Peroxisomal Proteins in Skeletal Tissues

To gently remove large amounts of paraffin, slides with sections were placed at 37°C for one week. Thereafter, they were deparaffinized with xylene (3 x 5 min) followed by rehydration in a series of ethanol (2 x 99%, 96%, 80%, 70%, 50% ethanol, 2 min each step). For improved antigen retrieval and accessibility of epitopes, deparaffinized and rehydrated decalcified skeletal tissue sections from adult mice were subjected to digestion with 0.1% trypsin for 20 min at 37°C and non-decalcified sections from newborn mice were incubated in buffer containing 5 mM EGTA and 0.1 M Tris (pH 9.0), in a microwave for 5–6 min. Non-specific binding sites were blocked with 4% BSA and 0.05% Tween 20 in PBS for 2 h at room temperature and sections were incubated with primary antibodies (Table 1) overnight at 4°C. The following morning, the sections were rinsed carefully with PBS and thereafter incubated with the secondary antibodies (Table 2) for 2 h at room temperature. Nuclei were labeled with Hoechst 33342 (2 μg/ml) or TOTO-3-iodide (1 μg/ml). Negative control sections without primary antibody incubation were processed in parallel.

### 5. *In Situ* Hybridization

*In situ* hybridization was performed as previously described by Grabenbauer et al. [22]. In brief, deparaffinized tissue sections were pretreated with 100 mM HCl, digested for 30 min at 37° C with proteinase K in a buffer containing 100 mM Tris, 50 mM EDTA (pH 8.0) and post-fixed for 5 min with 4% paraformaldehyde in PBS. Thereafter, tissue sections were incubated in 0.25% (v/v) acetic acid anhydride in 100 mM triethanolamine at pH 8.0, followed by dehydration in ethanol and air-drying. The sections were prehybridized for 2 h at 45°C in a mixture consisting of 50% (v/v) formamide, 50 mM Tris-HCl (pH 7.5), 25 mM EDTA, 20 mM NaCl, 250 mg/ml yeast tRNA, and 2,5 x Denhardt’s solution. Digoxigenin-labeled riboprobes for *Pex11ß* and their 200-base fragments were synthesized as described previously [23]. Each section was incubated overnight at 45°C with 20 μl of the hybridization mixture containing 5 ng/ml riboprobe, 50% (v/v) formamide, 20 mM Tris-HCl (pH 7.5), 1 mM EDTA, 333 mM NaCl, and 10% dextran sulfate. Corresponding negative controls were incubated in parallel with an mRNA sense probe for *Pex11ß*. Thereafter, the sections were washed at 53°C with 2 × SSC (standard saline citrate buffer: 300 mM NaCl, 30 mM sodium citrate, pH 7.2) for 30 min, thereafter at room temperature with 1 × SSC/50% (v/v) formamide for 1 h, 2 x with 0.5 × SSC for 10 min and 1x with 0.2 × SSC for 10 min. The hybridizations at 58°C were washed more stringent with 2 × SSC for 30 min at room temperature, followed by washing with 2 × SSC and 0.1 × SSC for 1 h at 65°C. Before digoxigenin detection, nonspecific binding sites were blocked with 1% (w/v) blocking medium (Boehringer Mannheim; Mannheim, Germany), and 0.5% (w/v) BSA in Tris-buffered saline (TBS; 100 mM Tris, 150 mM NaCl, pH 7.5). The sections were incubated overnight at 4°C with alkaline phosphatase-labeled anti-digoxigenin Fab fragments diluted in blocking buffer according to the manufacturer’s recommendation. The staining reaction for alkaline phosphatase was performed at 37°C in darkness with a buffer containing 100 mM Tris, 100 mM NaCl, 50 mM MgCl_2_ (pH 9.5), 275 mM nitroblue tetrazolium salt, 400 mM 5-bromo-4-chloro-3-indolyl phosphate, and 1 mM levamisole. Finally, the sections were counterstained with hematoxylin or nuclear Fast Red and mounted with glycerol–gelatin.

### 6. Isolation, Culture Conditions and Drug Treatment of Primary Osteoblasts and MC3T3-E1 cells

Primary osteoblasts were isolated from newborn pups. After decapitation, calvariae were removed and washed with ɑ-MEM. The fibrous tissue surrounding the bone was gently scraped off with a tweezer. The calvariae were divided into two halves and the sutures were cut out. The trimmed calvariae were transferred to a 50 ml Erlenmeyer flask containing 4 mM EDTA and placed in a shaking water bath (37°C) for 10 min, washed with PBS for 5 min, and incubated a second time in 4 mM EDTA at 37°C for 10 min. Calvariae were then subjected to a series of collagenase II digestions in a 37°C water bath with gentle shaking. The first two digests were discarded. Digests 3, 4, and 5 (15 min each), which were sufficient to release all cells from the small calvariae, were neutralized with ɑ-MEM, pooled, and filtered through a sterile mesh of 250 μm pore size (Reichelt GmbH Co KG, Haan, Germany) into a 50 ml tube. The filtrate was centrifuged for 5 min at 200 *g*, the supernatant was removed, and the cells were re-suspended in ɑ-MEM containing 10% FCS and antibiotics and seeded into 35 mm-diameter poly-L-lysine-coated culture dishes. The next morning the medium was exchanged and the cells were cultivated with regular medium exchange every two days. The purity of the culture (95% osteoblasts) and differentiation of the cells were analyzed by immunostaining for osteopontin (OPN) and osteocalcin as specific middle and late stage markers. In addition, we examined mineralization of osteoblasts after 7 and 15 days in culture by Alizarin red staining. This staining was performed by rinsing the cells with PBS followed by incubation with 95% ethanol for 15 min at room temperature. Next, cells were washed with distilled water and the formed calcium nodules were stained with 0.1% Alizarin Red S in 1% Tris-HCl (pH 8.3) for 40 min at 37°C. Colored culture dishes were air-dried and scanned with an Epson perfection 1660 photo scanner.

In some series of experiments, we used non-transformed MC3T3-E1 mouse calvarial fibroblasts (established from the calvaria of a C57BL/6 mouse embryo/fetus) which were described to differentiate into osteoblasts [24,25]. Cells were cultured in ɑ-MEM containing 10% FCS and antibiotics and were passaged before confluence.

For drug treatment, primary osteoblasts and MC3T3-E1 cells were cultured for 3 days after isolation or passaging, were then trypsinized and re-seeded with a recovery period of 24 h followed by a 6 day-treatment with either the PPARɑ agonist ciprofibrate (100 μM, 500 μM), the PPARɑ antagonist GW6471 (10 μM), the PPARß agonist GW0742 (30 μM), the PPARß antagonist GSK0660 (150 nM), the PPARɣ agonist troglitazone (2 μM, 10 μM) or the irreversible PPARɣ antagonist GW9662 (40 μM). Drugs for activation or inhibition of the different PPARs used in this study followed the actual pharmacology guide for nuclear hormone receptors [26]. Drug concentrations were chosen with regard to their EC50 values and were validated for their toxicity (MTT assay) in calvarial osteoblasts (ciprofibrate: toxic >800 μM, GW6471: not toxic up to 50 μM, GW0742 toxic >110 μM, GSK0660: not toxic up to 250 nM, troglitazone: toxic >10 μM, GW9662: not toxic up to 50 μM). Every 2 days, fresh medium containing the same amount of drug or vehicle (0.1% DMSO) was added.

### 7. Indirect Immunofluorescence on Primary Osteoblasts

Primary osteoblasts grown on poly-L-lysine-coated coverslips were rinsed with PBS and fixed with 4% paraformaldehyde in PBS for 20 min at room temperature. After fixation, cells were washed three times with PBS. Thereafter, they were incubated for 10 min in PBS containing 1% glycine and for an additional 10 min in PBS containing 1% glycine and 0.3% Triton X-100 for permeabilization. After washing with PBS, cells were incubated for blocking of nonspecific protein binding sites for 30 min in PBS containing 1% BSA and 0.05% Tween 20. After blocking, the coverslips were incubated with primary antibodies overnight at 4°C (Table 1), followed by extensive washing with PBS (3 x 5 min) and incubation with secondary antibodies (Table 2) for 1 h at room temperature. Nuclei were counterstained with Hoechst 33342 (2 μg/ml) or TOTO-3-iodide (1 μg/ml). Images were saved in a tif-format and imported in Photoshop CC.

### 8. Analysis of the Numerical Abundance of Peroxisomes in Osteoblasts

Osteoblasts were stained after various time points of cultivation (3, 7, 11 and 15 d) with a rabbit anti-mouse PEX14 antibody (Table 1) and a donkey anti-rabbit Alexa Fluor 488 secondary antibody (Table 2) for analysis of numerical peroxisome abundance. For each time point, images from 50 osteoblasts were taken by confocal laser scanning microscopy. The number of peroxisomes per μm² was counted in each cell by using the Image-Pro Plus® program (Media Cybernetics, USA). For automatic quantification of the number of spots, a self-defined threshold was set at which peroxisomes could clearly be distinguished from background staining. All experiments were performed in triplicates.

### 9. Analysis of the Osteoblast Proliferation at Different Time Points

To quantify osteoblast proliferation at different time points in culture (3, 7, 11 and 15 d), the expression of Ki67 was used as a marker for S, G2, and M phases. Osteoblasts were stained with the rat anti-mouse Ki67 antibody (Table 1) and a goat anti-rat Alexa Fluor 594 secondary antibody (Table 2). For each time point, the number of Ki67-positive cells in comparison to the total number of cells (stained with the nuclear dye Hoechst 33342) was counted under a regular fluorescence microscope in 10 different areas and each area containing approximately 100–150 cells. All morphometric experiments were performed in triplicates. Stained preparations were analyzed either with a regular Leica DRMD fluorescence microscope equipped with a DC480 camera or with a Leica TCS SP2 confocal laser scanning microscope using 40x or 63x objectives and Airy1.

### 10. RNA Isolation followed by Semiquantitative and Quantitative RT-PCR

Total RNA was isolated from primary osteoblasts and MC3T3-E1 cells using the RNeasy Mini kit. First-strand cDNA was synthesized from DNAse I-treated 1.0 μg total RNA with oligo (dT) 12–18 primers using superscript II reverse transcriptase. For semiquantitative RT-PCR, the PCR reaction mix contained the template cDNA, 10 mM dNTPs, the 5 PRIME Taq DNA polymerase, and the 5 PRIME Master Mix. PCR reaction was performed in the Bio-Rad iCycler C1000 (Bio-Rad Laboratories, München, Germany) with the following parameters: denaturation at 95°C for 2 min; followed by 32–45 cycles of denaturation at 95°C for 30 sec, annealing at 50–65°C for 1 min, extension at 72°C for 1 min; and a final extension at 72°C, 7 min. Reaction products were then separated on 2% agarose gels, stained with SYBR® Gold and photographed using the Gel-Doc 2000 documentation system from Bio-Rad Laboratories (München, Germany).

For quantitative RT-PCR, we used the qPCR Maxima SYBR Green Master Mix which was mixed 1:1 with the template cDNA and the forward and reverse primer. All samples were run in triplicates in each of 3 different series of experiments. The PCR reaction was done in the IQ5 iCycler (Bio-Rad Laboratories, München, Germany) using the following 3-step amplification protocol: 2 min at 95°C (enzyme activation), 42 cycles of 15 sec at 95°C (denaturation), 30 sec at 60°C or 65°C (annealing) and 30 sec at 72°C (extension). All primer pairs for semiquantitative (Table 3) and quantitative RT-PCR (Table 4) were designed using the PRIMER3 program (http://www.ncbi.nlm.nih.gov/tools/primer-blast). Primer pairs for quantitative RT-PCR were verified for specificity showing a single peak in the melting curve analysis as well as for their amplification efficiency by 10-fold dilutions series (efficiency coefficients are given in Table 4). Calculation of the relative gene expression was done by the 2^-∆∆ct^ method [27] using *Actb* or *Gapdh* as reference gene.

**Table 3:** List of primer pairs for semi-quantitative RT-PCR.

*Gene*	Refseq Accession#		5´- 3´ Sequence	Size	Temp	Cycles
*Abcd1*	NM_007435.1	F	GAGGGAGGTTGGGAGGCAGT	465	65	35
* *		R	GGTGGGAGCTGGGGATAAGG			
*Abcd3*	NM_008991.2	F	CTGGGCGTGAAATGACTAGATTGG	523	64	35
* *		R	AGCTGCACATTGTCCAAGTACTCC			
*Acox1*	NM_015729.3	F	CTGAACAAGACAGAGGTCCACGAA	565	60	35
* *		R	TGTAAGGGCCACACACTCACATCT			
*Acox2*	NM_053115.2	F	CTCTTGCACGTATGAGGGTGAGAA	688	60	35
* *		R	CTGAGTATTGGCTGGGGACTTCTG			
*Alp*	NM_0012871722	F	GCCCTCTCCAAGACATATA	373	55	33
* *		R	CCATGATCACGTCGATATCC			
*Cat*	NM_009804.2	F	ATGGTCTGGGACTTCTGGAGTCTTC	833	64	40
* *		R	GTTTCCTCTCCTCCTCATTCAACAC			
*Gapdh*	NM_008084	F	CACCATGGAGAAGGCCGGGG	391	60	28
* *		R	GACGGACACATTGGGGGTAG			
*Mfp1*	NM_023737.3	F	ATGGCCAGATTTCAGGAATG	211	60	35
* *		R	TGCCACTTTTGTTGATTTGC			
*Mfp2*	NM_008292	F	GAGCAGGATGGATTGGAAAA	213	60	35
* *		R	TGACTGGTACGGTTTGGTGA			
*Opn*	NM_001204208.1	F	TCACCATTCGGATGAGTCTG	437	58	28
* *		R	ACTTGTGGCTCTGATGTTCC			
*Ppara*	NM_011144.6	F	AGACCGTCACGGAGCTCACA	584	68	35
* *		R	GGCCTGCCATCTCAGGAAAG			
*Pparb*	NM_011145.3	F	CACCGAGTTCGCCAAGAACA	363	60	35
* *		R	AGAGCCCGCAGAATGGTGTC			
*Pparg*	NM_001127330.1	F	TCCGTAGAAGCCGTGCAAGA	441	60	35
* *		R	CACCTTGGCGAACAGCTGAG			
*Pex5*	NM_008995.2	F	GAGTGAAGAAGCAGTGGCTGCATAC	508	64	30
* *		R	GGACAGAGACAGCTCATCCCTACAA			
*Pex11a*	NM_011068.1	F	TGCTTAGATATTTGTTAGAG	420	64	35
* *		R	GTACTTAGGAGGGTCCCGAGAGGA			
*Pex11b*	NM_0011069	F	GTATGCCTGTTCCCTTCTCG	216	65	35
* *		R	CTCGGTTGAGGTGACTGACA			
*Pex11g*	NM_026951.2	F	GACTCTGCTTGGTGGTGGACACT	682	64	35
* *		R	TGTCTCTCCCACTCACCTTTAGGC			
*Pex13*	NM_023651.4	F	GACCACGTAGTTGCAAGAGCAGAGT	718	65	35
* *		R	CTGAGGCAGCTTGTGTGTTCTACTG			
*Pex14*	NM_019781.2	F	CACCTCACTCCGCAGCCATA	131	60	35
* *		R	AGGATGAGGGGCAGCAGGTA			
*Runx2*	NM_00146038.2	F	CCGCACGACAACCGCACCAT	289	62	35
* *		R	CGCTCCGGCCCACAAATCTC			

**Table 4:** List of primer pairs for quantitative RT-PCR.

*Gene*	Refseq Accession#		5´- 3´ Sequence	Size	Eff
*Acox1*	NM_015729.3	F	CCGCCACCTTCAATCCAGAG	86	1,99
		R	CAAGTTCTCGATTTCTCGACGG		
*Actb*	NM_007393.3	F	GCTCCTCCTGAGCGCAAG	75	1,99
		R	CATCTGCTGGAAGGTGGACA		
*Alp*	NM_0012871722	F	AGGGCAATGAGGTCACATCC	80	1,96
		R	CACCCGAGTGGTAGTCACAAT		* *
*Oc*	NM_001037939.2	F	TGACCTCACAGATGCCAAGC	93	1,79
		R	CGCCGGAGTCTGTTCACTAC		
*Cat*	NM_009804.2	F	GGAGGCGGGAACCCAATAG	102	1,99
		R	GTGTGCCATCTCGTCAGTGAA		
*Col1a1*	NM_007742.3	F	GCTCCTCTTAGGGGCCACT	91	1,99
		R	ATTGGGGACCCTTAGGCCAT		
*Gapdh*	NM_008084	F	TGGCAAAGTGGAGATTGTTGCC	156	1,99
		R	AAGATGGTGATGGGCTTCCCG		
*Pex11a*	NM_011068.1	F	ACTGGCCGTAAATGGTTCAGA	119	1,99
* *		R	CGGTTGAGGTTGGCTAATGTC		
*Pex11b*	NM_0011069	F	CGCCTATTGATGGAACAAGAGACT	96	1,99
* *		R	TCCAGGTCCCACAGTTTCTACTC		
*Pex11g*	NM_026951.2	F	CTAGTGGAACAATGCCCCAAC	137	1,89
* *		R	AGGCCATACTGCTTAGTGTAGA		
*Pex13*	NM_023651.4	F	TGGATATGGAGCCTACGGAAA	81	1,99
		R	CGGTTAAAGCCCAAACCATTG		
*Pex14*	NM_019781.2	F	GCCACCACATCAACCAACTG	97	1,99
		R	GTCTCCGATTCAAAAGAAGTCCT		
*Ppara*	NM_011144.6	F	AGACCCTCGGGGAACTTAGA	123	1,89
		R	CAGAGCGCTAAGCTGTGATG		
*Pparb*	NM_011145.3	F	GCAGCCTCAACATGGAATGTC	96	1,99
		R	GAGCTTCATGCGGATTGTCC		
*Pparg*	NM_001127330.1	F	TTTTCAAGGGTGCCAGTTTC	112	1,99
		R	CATGGACACCATACTTGAGCA		
*Runx2*	NM_00146038.2	F	CGGTGCAAACTTTCTCCAGGA	105	1,83
* *		R	GCACTCACTGACTCGGTTGG		
*Opn*	NM_001204208.1	F	GGTCAAAGTCTAGGAGTTTCCAG	87	1,96
* *		R	CACCGCTCTTCATGTGAGAGG		

### 11. Isolation of Whole Cell Homogenates and of Mitochondrial and Enriched Peroxisomal Fractions from Primary Osteoblasts

To obtain whole cell homogenates from primary osteoblasts), cells were rinsed with PBS and suspended with 10 volumes of ice-cold lysis buffer containing 50 mM Tris-HCl (pH 7.2), 250 mM NaCl, 0.1% NP-40, 2 mM EDTA, 10% glycerol and 1% protease inhibitor mix M. Incubation was done on a rotating shaker for 30 min at 4°C. Thereafter, the cells were lysed by a single sonication for 10 sec. Finally, the tube was centrifuged at 12,000 *g* for 10 min at 4°C to remove non-lysed cells and cell debris and the supernatant was collected for experiments.

For analyzing proteins of the mitochondrial and peroxisomal compartment, differential fractionation of primary osteoblasts was performed. Cells were collected using a cell scraper and were homogenized with a single stroke (2 min, 1000 rpm) using a Potter-Elvehjem homogenizer (Braun, Melsungen, Germany) in homogenization medium (HM: 5 mM MOPS, pH 7.4, 250 mM sucrose, 1 mM EDTA, 0.1% [v/v] ethanol, 0.2 mM dithiothreitol, 1 mM 6-aminocapronic acid) supplemented with 10% protease inhibitor mix M. The homogenate was centrifuged at 500 *g* for 10 min. The resulting supernatant (S1a) was kept on ice, and the pellet was resuspended in HM and centrifuged at 500 *g* for 10 min at 4°C, resulting in the supernatant (S1b) and the pellet (P1) with unbroken cells and nuclei. The pooled supernatant S1 (S1a + S1b) was further subjected to centrifugation at 1,900 *g* for 10 min, the supernatant (S2a) was collected and kept on ice, and the pellet was dissolved in HM and centrifuged at 1,900 *g* for 10 min at 4°C, resulting in the supernatant (S2b) and the pellet (P2) with heavy mitochondria. The pooled supernatant S2 (S2a + S2b) was centrifuged at 50,000 *g* for 20 min to yield the peroxisomal fraction (pellet) and the supernatant (S3a). The peroxisomal pellet was resuspended in HM and centrifuged at 50,000 *g* for 20 min, yielding the enriched peroxisomal fraction (P3) and the supernatant (S3b). The supernatant S3a plus S3b were combined (microsomal and cytosolic fraction S3). Fractions S2, P2, S3, and P3 were analyzed by Western blotting. The enriched peroxisomal fraction is a mixed organelle fraction, also known as light mitochondrial fraction (LM) or D-fraction, containing a high amount of medium sized peroxisomes as well as small mitochondria, lysosomes, and a small amount of microsomal vesicles.

### 12. Western Blot Analysis

Protein concentrations of the whole cell homogenates and of the isolated subcellular fractions were determined in triplicates with the Bradford method using BSA as a standard [28]. Equal amounts of protein (30 μg in case of whole cell homogenates, 7–10 μg in case of the fractions S2, S3, P2, P3) were loaded onto 12% SDS polyacrylamide gels. After electrophoresis, the transfer of equal amounts of proteins onto polyvinylidene difluoride membranes was controlled by Ponceau staining. Nonspecific protein-binding sites were blocked with TBS containing 10% non-fat milk powder and 0.05% Tween 20 for 1 h. The blots were incubated overnight at 4°C or for 1 h at room temperature with primary antibodies (Table 1). Thereafter, the membranes were washed 3 x 10 min at room temperature and were then incubated for 1 h with alkaline phosphatase (ALP)-conjugated secondary antibodies (Table 2). Chemiluminescence detection of alkaline phosphatase activity was done using the Immun-Star™ ALP substrate and exposure of the blots to Kodak Biomax MR Films (Sigma-Aldrich, Deisenhofen, Germany). All Western blots were scanned using an Agfa Horizon Ultra Color Scanner (Agfa, Mortsel, Belgium) and imported into Photoshop CC. Semiquantitative analysis of the integrated optical densities of the bands was performed using the QuantityOne® software program (Bio-Rad Laboratories, München, Germany). To compare protein levels under different conditions, we used ɑ-tubulin as reference protein for the whole cell homogenates. In case of the isolated subcellular fractions, comparability was ensured by loading the same amount of protein in each lane [29].

### 13. Dual-Luciferase Reporter Gene Assay

The PPRE luciferase reporter assay experiments were performed using the Dual-Luciferase Reporter Assay System together with a PPAR Signal Reporter Kit from Qiagen according to the protocol of the manufacturer. Briefly, MC3T3-E1 cells grown on a 6-well plate were transfected with the PPRE vector or the negative control vector using 4 μl Trans IT® LT-1 transfection Reagent (Cat. MIR2300) purchased from Mirus (through VWR, Darmstadt, Germany) according to the instructions of the manufacturer. The luciferase activity [30] was measured 48 h after transfection with the luminometer Lumat LB 9507 from BERTHOLD Technologies, Pforzheim, Germany.

### 14. Statistical Analysis

Significant differences between the mean values of non (vehicle)-treated controls versus drug-treated groups were analyzed using one-way ANOVA test followed by post-hoc Scheffé-test with *p≤0.05; **p≤0.01; ***p≤0.001. Data are presented as the mean ± standard deviation (SD).

## Results

In this study, we characterized peroxisomal distribution and function in bone and cartilage during intramembranous and endochondral ossification since defects in these processes are typical features and hallmarks of human patients with different peroxisomal biogenesis disorders. In addition, we analyzed the role of peroxisomes and PPARs during osteoblast differentiation.

### 1. Peroxisomes are Present with Heterogeneous Abundance in Different Cell Types of Bone and Cartilage

**Fig 1:**
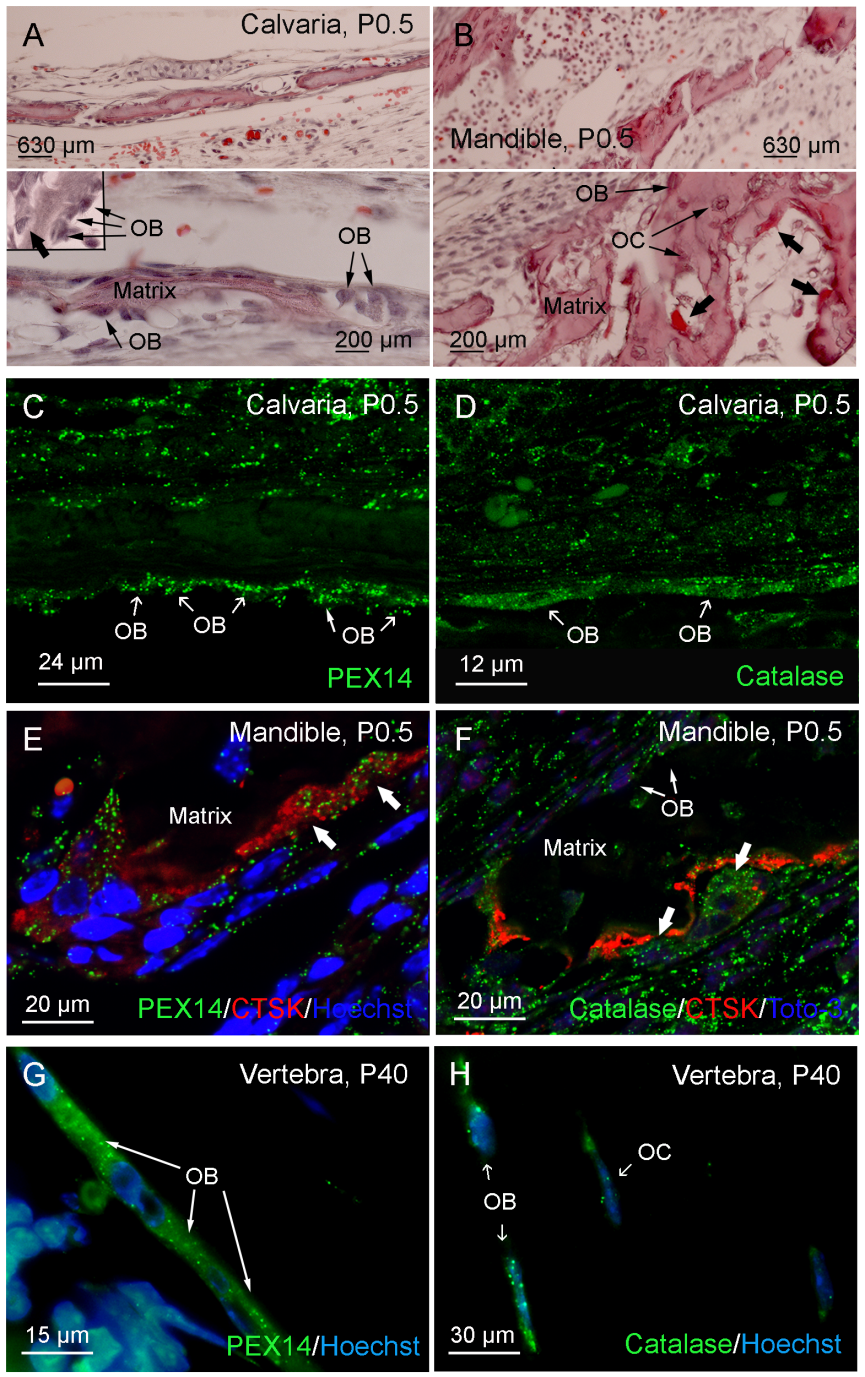
The highest abundance of peroxisomes was detected in osteoblast and osteoclast cells as examples for intramembranous and endochondral ossification. (A-B) Paraffin sections of the calvaria (A) and mandible (B) of newborn mice (P0.5) were stained with hematoxylin and eosin to give an overview on bone architecture and localization of osteoblasts (OB), osteocytes (OC) and osteoclasts (bold arrows). (C-H) Immunofluorescence stainings for PEX14 (C, E, G) and catalase (D, F, H) were performed in paraffin sections from the calvaria (C, D) and mandible (E, F) of newborn mouse and vertebrae of adult mice (G, H) Bold arrows in E and F indicate cathepsin K (CTSK)-positive osteoclasts. Please note the higher abundance of peroxisomes in osteoblasts and osteoclasts than in osteocytes as well as in adult (P40) compared to newborn mice (P0.5).

**Fig 2:**
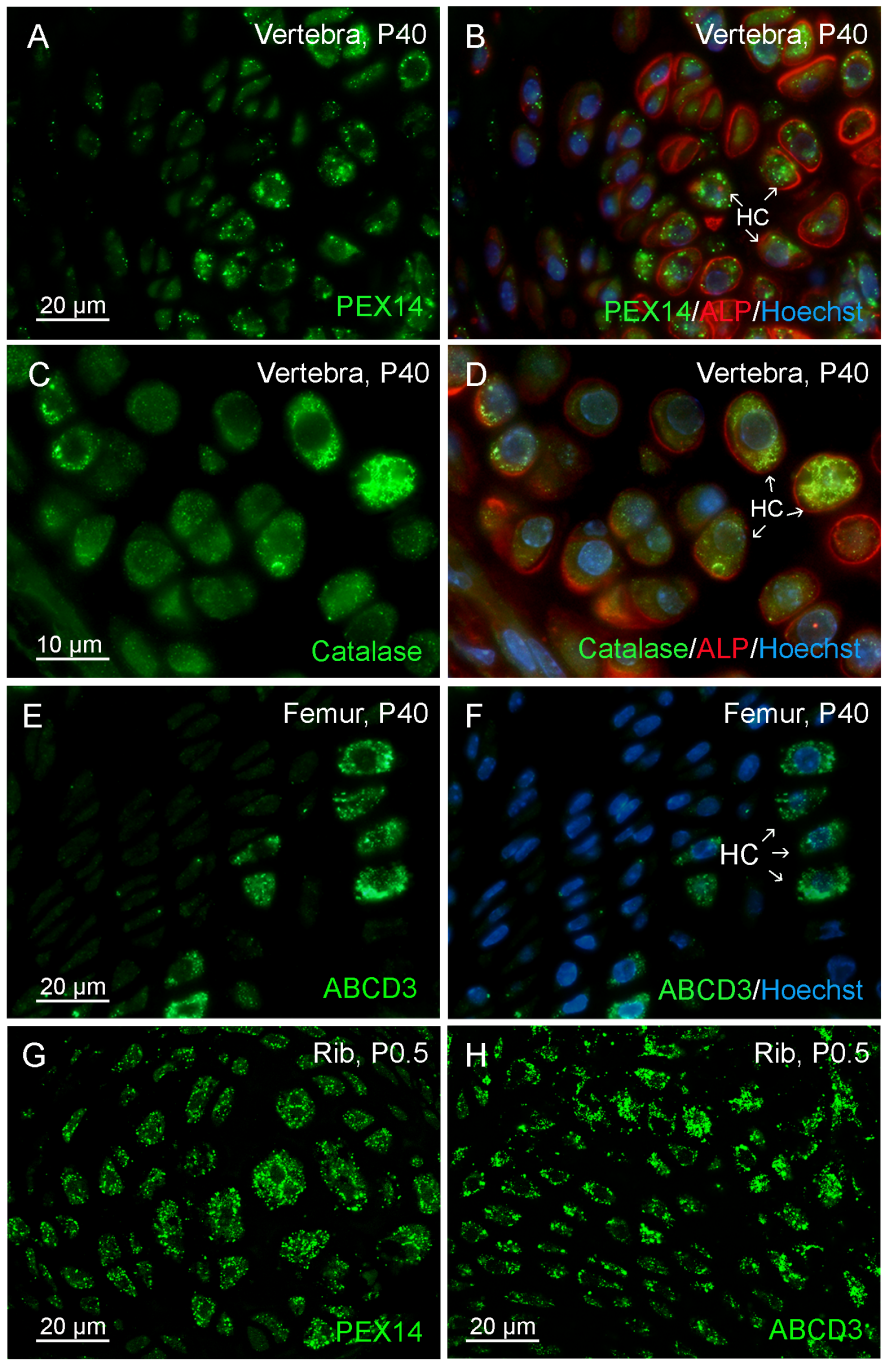
Hypertrophic chondrocytes contained the highest numerical abundance of peroxisomes compared to proliferative chondrocytes as examples for endochondral ossification. (A-H) Immunofluorescence stainings of the peroxisomal membrane and matrix proteins PEX14 (A, B, G), catalase (C, D, H) and ABCD3 (E, F) were performed in paraffin-sections from the cartilage (A-D: vertebrae; E, F: femur growth plate; G, H: ribs) of 40-day (A-F) and P0.5 newborn (G, H) mice. Nuclei were stained with Hoechst 33342 (Hoechst) or TOTO-3-iodide (TOTO-3); ALP immunoreactivity (B, D) was used as a marker for skeletal tissue. HC = hypertrophic chondrocytes.

Since the peroxisomal biogenesis protein PEX14 is an ideal marker for the detection of peroxisomes independent of their metabolic activity [31], immunofluorescence preparations for PEX14 in comparison to the metabolic proteins catalase and ABCD3 were used to analyze the distribution of peroxisomes in the distinct cell types during intramembranous (Fig 1) and endochondral (Fig 2) ossification. An overview on bone architecture is given for the calvaria (Fig 1A) and mandible (Fig 1B) to localize osteoblasts (OB, cubic to rectangular cells on the surface of the bone matrix), osteoclasts (bold arrows, sitting attached with their ruffled borders inside the Howship´s lacunaes), and osteocytes (OC, surrounded by the bone matrix, laying inside small lacunae’s). Osteoblasts of the calvaria (Fig 1C and 1D) and the mandible (Fig 1E and 1F) as examples for intramembranous ossification and of the vertebrae (Fig 1G and 1H) were intensively labelled for PEX14 (Fig 1C, 1E and 1G) and catalase (Fig 1D, 1F and 1H) revealing a high number of peroxisomes in this cell type. Interestingly, also osteoclasts showed a high numerical peroxisome abundance in PEX14 (Fig 1E) and catalase (Fig 1F) immunofluorescence stainings. In contrast, osteocytes contained only a few weakly stained peroxisomes (Fig 1H). In endochondral ossification, osteoblasts in the ossification center and in the periostal cartilage collar were similarly strong labelled as the ones in intramembranous ossification (not shown). The number and size of peroxisomes in chondrocytes constantly increased from the reserve to the proliferative and hypertrophic zone (Fig 2A, 2B and 2G). These cell-type specific differences were even more pronounced in immunofluorescence stainings for the peroxisomal matrix enzyme catalase (Fig 2C and 2D) and the peroxisomal membrane transporter ABCD3 (Fig 2E, 2F and 2H).

### 2. Pex11ß Gene Expression Corroborates the Results on the Numerical Abundance of Peroxisomes in Distinct Cell Types of the Skeleton

**Fig 3:**
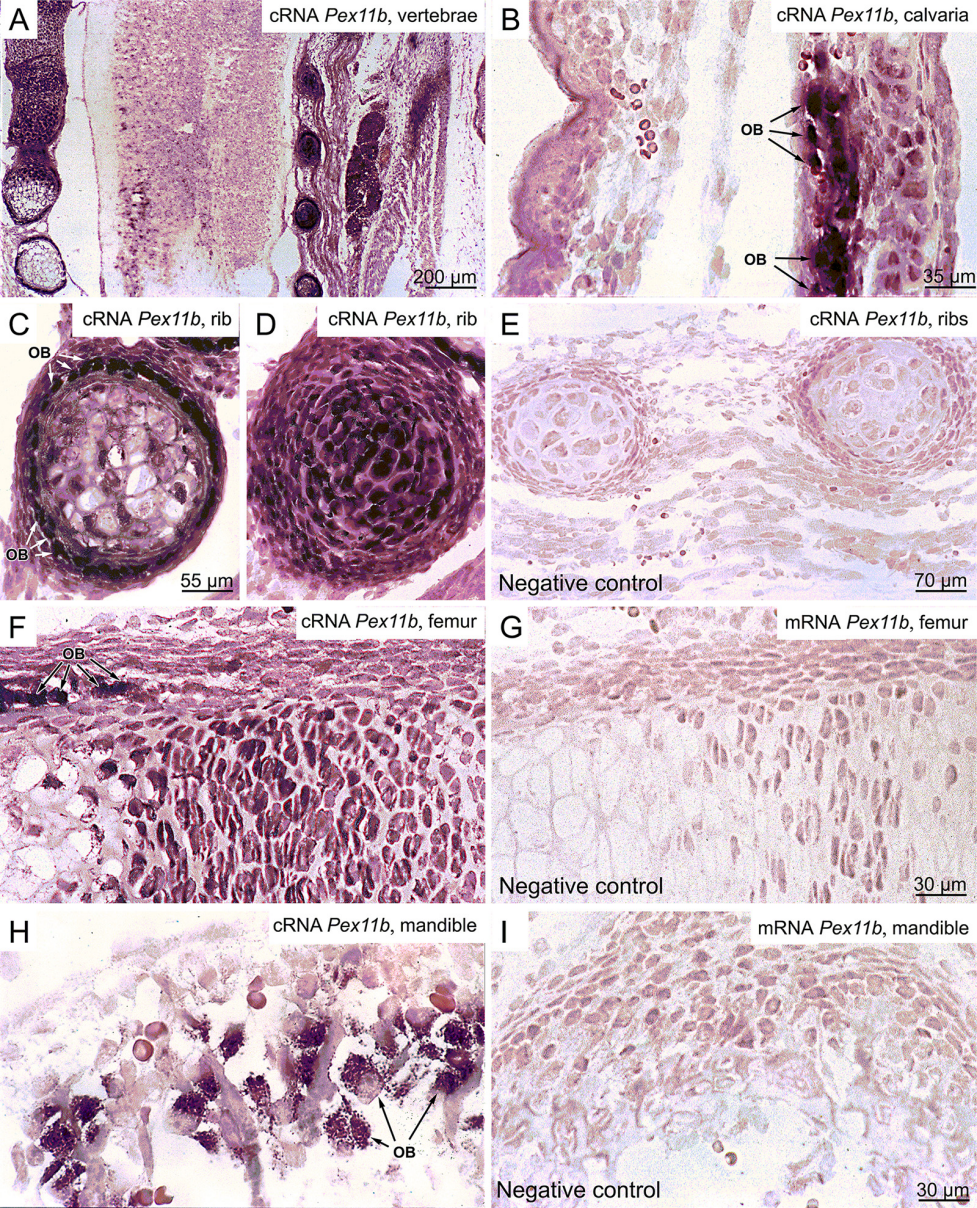
The distribution of *Pex11ß* cRNA revealed the strongest expression in the mineralization areas of the cartilage and in osteoblasts of the calvaria of newborn mice. (A-H) Higher magnifications of *in situ* hybridization preparations of *Pex11ß* cRNA in vertebrae (A), the calvaria (B), ribs (C, D), femur (F, G), and the mandible (H) are shown. The corresponding negative controls were hybridized with the complementary *Pex11ß* mRNA strand (I). Please note that the expression of *Pex11ß* cRNA in the calvariae showed a higher level in osteoblasts than in osteocytes. OB: osteoblasts.

The constitutive number of peroxisomes in distinct cell types is generally regulated by the PEX11ß protein level. Unfortunately the capability of the currently commercially available antibodies is insufficient for morphological analysis of this protein in paraffin sections. Therefore, we performed *in situ* hybridization for the localization of its mRNA. Already at lower magnification of *in situ* hybridization preparations of paraffin sections of newborn pups, it became evident that bone and cartilage exhibited the highest level of *Pex11ß* mRNA expression in comparison to neighboring tissues such as connective tissue, skeletal muscle, nervous tissue, and epithelial cells of the skin (Fig 3A and 3B). Only adipose tissue was nearly as strong stained for *Pex11ß* mRNA (Fig 3A). The highest *Pex11ß* mRNA expression was found in osteoblasts of both types of ossification processes (Fig 3B—calvaria, Fig 3C—rib, Fig 3F—femur, and Fig 3H—mandible). In chondrocytes, the *Pex11ß* mRNA expression varied depending on their stage during endochondral ossification (Fig 3A–3D, 3F and 3H); large hypertrophic chondrocytes were lost during to the protease and microwave treatment of thin (2 μm) paraffin sections of the *in situ* hybridization (Fig 3C, 3F and 3G). For all cell types, a clear positive correlation of the *Pex11ß* mRNA expression with the numerical abundance of peroxisomes was observed corroborating the data obtained in immunofluorescence preparations for PEX14 (Figs 1 and 2). The high specificity of our hybridization procedure is well documented by the negative nuclei even in the strongest *Pex11ß* cRNA-labeled osteoblasts (Fig 3H) as well as in corresponding *Pex11ß* mRNA negative controls which were always devoid of labeling (Fig 3E, 3G and 3I).

### 3. Peroxisome Numerical Abundance Parallels Osteoblast Differentiation in Cell Culture

**Fig 4:**
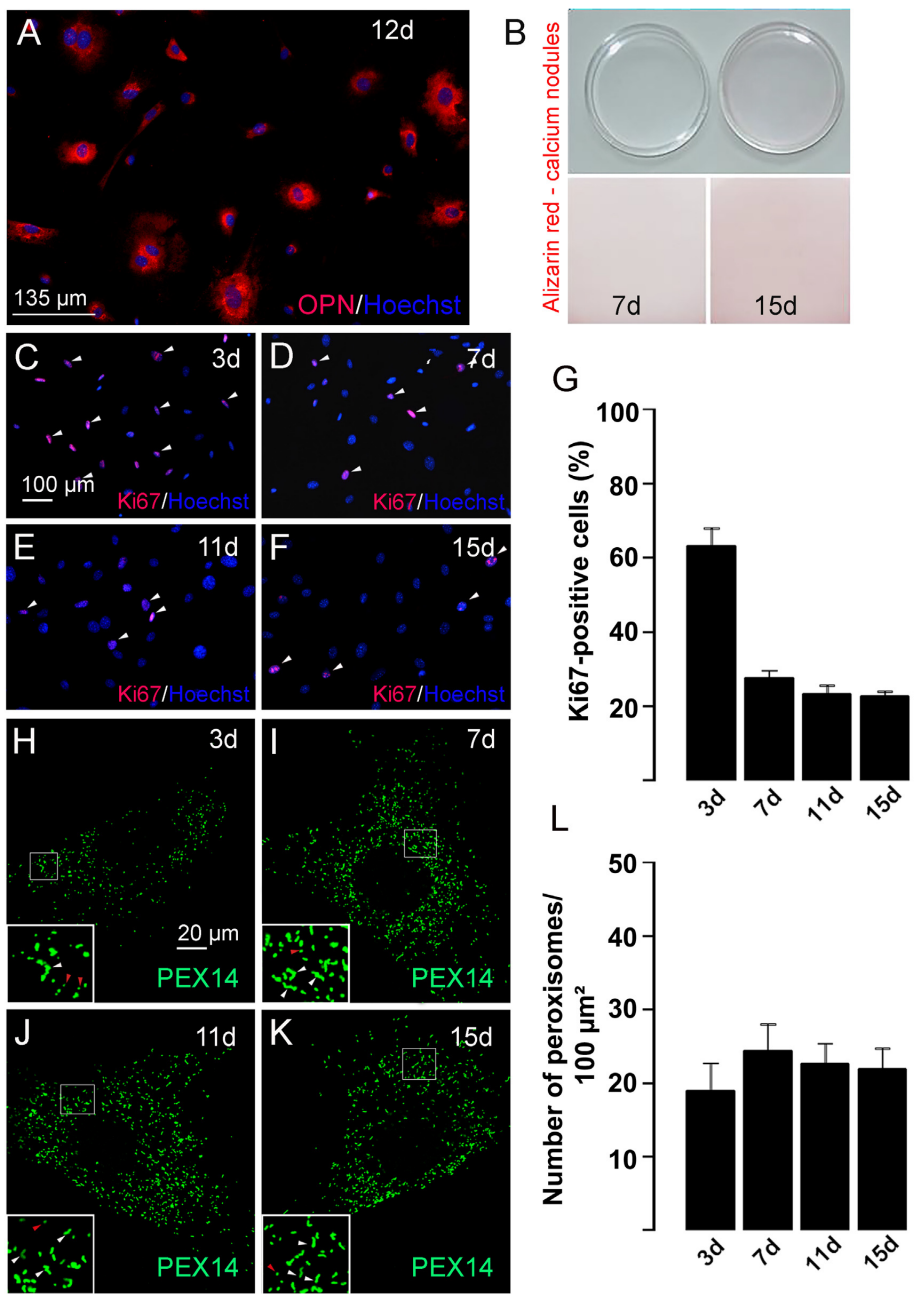
The peroxisome numerical abundance increased during osteoblast differentiation. (A) In primary osteoblast cultures (day 12) from the calvariae of newborn mice, more than 95% of the cells were positively stained for the early osteoblast marker OPN. (B) The formation of calcium nodules increased in primary osteoblasts during culture as shown by Alizarin red staining of the Petri dishes. (C-L) Immunofluorescence stainings of osteoblasts at different time points during culture detecting cell proliferation using anti-Ki67 antibodies (C-G) and peroxisome numerical abundance using anti-PEX14 antibodies (H-L). Nuclei were visualized with Hoechst 33342 (Hoechst, C-F). Representative images (C-F, H-K) and quantitative analysis (G, L) are shown.

**Fig 5:**
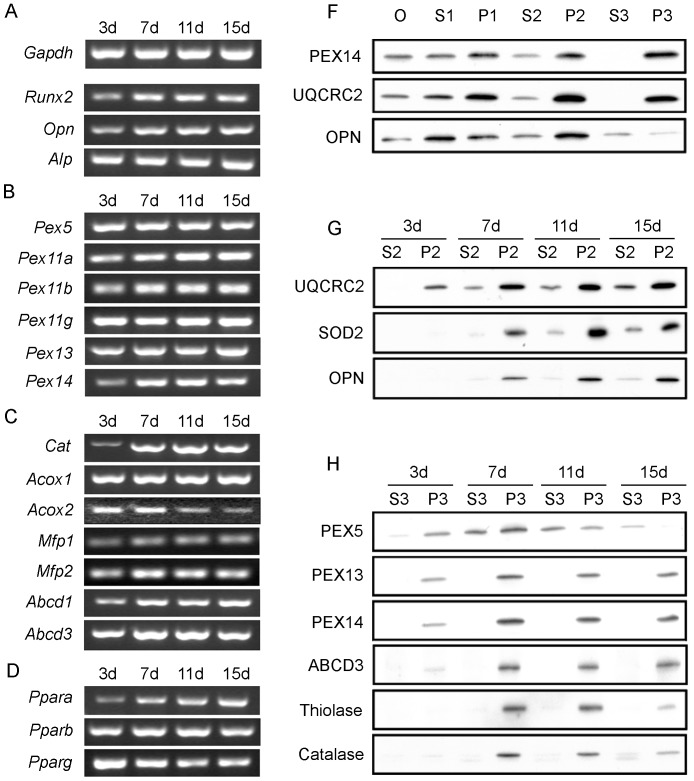
Osteoblast differentiation is accompanied by increases in the expression of peroxisome-related genes and proteins. (A-D) Semiquantitative RT-PCR of genes involved in osteoblast differentiation (A), peroxisome biogenesis (B) as well as of peroxisomal enzymes and transporters (C) and *Ppars* (D). The mRNA level of the housekeeping gene *Gapdh* is included in (A). G-H. Increases in the protein level of peroxisomal membrane and matrix proteins during osteoblast differentiation were confirmed by Western blot analyses of organelle fractions from primary osteoblasts. (F) Osteoblasts after 15 days in culture were collected and subjected to differential centrifugation to obtain enriched organelle fractions (S2, P2, S3, and P3: all the details can be found in Methods, chapter 11). Fractionation quality is demonstrated by Western blots for the peroxisomal marker protein PEX14, mitochondrial marker protein UQCRC2, and the cytosolic, extracellular and vesicular marker protein OPN. (G-H) Time-dependent changes in the protein levels of mitochondrial proteins SOD2, UQCRC2 (G), the bone maturation marker OPN (G) and of peroxisomal membrane (PEX13, PEX14, ABCD3) and matrix (Thiolase, Catalase) proteins (H) are shown.

Since Zellweger syndrome patients exhibit mostly skull defects due to alterations in intramembranous ossification, next experiments focused on osteoblast differentiation. For this purpose, we used primary cultures prepared from the calvaria of newborn mice. These cultures contained more than 95% osteoblasts as detected by a positive staining for the early bone marker protein OPN at day 12 in culture (Fig 4A). Cells differentiated from pre-osteoblasts to mature osteoblasts since we found 30-fold higher mRNA levels for OPN (early to middle stage marker) than for the early stage markers ALP and collagen 1ɑ1. In addition, among the late stage markers (e.g. osteocalcin, bone sialoprotein, dentin mineralization protein 1 [32,33]), osteocalcin and OPN were found to be expressed in all osteoblasts, but with heterogeneous immunoreactivities (Fig 4A). Furthermore, an increasing number of calcium deposits were detectable when the cells were cultured up to 15 days (Fig 4B) indicating matrix mineralization and maturation of the cells. To relate the alteration of the peroxisomal compartment to osteoblast proliferation, we analyzed the Ki67 protein expression, as a marker of late G1, S, G2, and M phases. Immunofluorescence preparations for Ki67 revealed that osteoblasts exhibited the highest proliferation rate at day 3 (63 ± 4.8%) (Fig 4C and 4G). In the period between day 3 and day 7, their proliferation rate decreased strongly and is only minor reduced thereafter (7 days: 27.6 ± 2%; 11 days: 23.3 ± 2.3%; 15 days: 22.7 ± 1.1%; Fig 4D–4H) which is in parallel to the increase in OPN, a marker for osteoblast maturation (Fig 5A and 5G). Interestingly, the most significant alterations in the numerical abundance and shape of peroxisomes were also noted within this period. In the early osteoblasts (3d), the number of peroxisomes was lowest (18.9 ± 3.7 peroxisomes/100 μm^2^, Fig 4H and 4L) and the majority of peroxisomes were spherical with only a few exhibiting a tubular structure (Fig 4H). At later time points (7, 11, and 15 days), osteoblasts contained a higher abundance of peroxisomes with a peak at day 7 (24.4 ± 3.6 peroxisomes/100 μm^2^; Fig 4I and 4L). Moreover, more tubular peroxisomes were detected from day 7 onwards (Fig 4J–4L).

### 4. Peroxisome-related Genes and Proteins are Heterogeneously Expressed during Osteoblast Differentiation

The results from above indicate that peroxisomal compartment in osteoblasts is dependent on their differentiation state. Therefore, primary osteoblasts in culture were again used to determine the expression levels of mRNAs encoding peroxisomal proteins at distinct differentiation time points (day 3, 7, 11 and 15) using semiquantitative RT-PCR. First, we analyzed the mRNAs encoding marker proteins for osteoblast differentiation (Fig 5A). Consistent with the decrease in cell proliferation and increase in osteoblast differentiation from day 3 up to day 7, the mRNA levels of the transcription factor *Runx2* and the secretory protein *Opn* were both up-regulated (Fig 5A). The mRNA levels of alkaline phosphatase (*Alp*), a marker for pre-osteoblasts during differentiation up to the maturation state [34] was not significantly changed (Fig 5A). With respect to the peroxisomal biogenesis proteins, the mRNA levels for PEX11ɑ and PEX11ß (peroxins responsible for peroxisome proliferation) as well as the one for PEX14 increased upon differentiation with a peak at day 7 (Fig 5B) corroborating the strong staining of the *Pex11ß* mRNA in osteoblasts in Fig 3. The mRNAs encoding peroxisomal membrane transporters (ABCD1, ABCD3) and multifunctional proteins 1 and 2 (MFP1, MFP2) were up-regulated between day 3 and 7 and remained stable thereafter, except for the mRNA for catalase, which peaked at day 7 to 11 and the one for acyl-CoA oxidase 2 (ACOX2), which was down-regulated after day 5 (Fig 5C). Many genes for peroxisomal proteins are regulated by transcription factors of the PPAR family, of which PPARß also influenced osteoblast differentiation [20]. Therefore, we analyzed the mRNAs levels for PPARɑ, PPARß and PPARɣ which were found to be differentially regulated during osteoblast differentiation. Whereas the *Pparɑ* mRNA levels were strongly increased between day 3 and 15, the ones for *Pparɣ* exhibited the opposite regulation and a strong decrease (Fig 5D). The mRNA level of PPARß slightly increased with a peak at day 7 (Fig 5D).

Moreover, we analyzed changes in the protein levels of distinct peroxisomal proteins during osteoblast differentiation in comparison to the mitochondrial marker SOD2 and the osteoblast differentiation marker OPN. Peroxisomal proteins exhibited the highest enrichment in pellet P3 (enriched peroxisomal fraction of intermediate sized organelles such as light mitochondria, medium sized peroxisomes, lysosomes, and a small amount of microsomal vesicles), whereas in supernatant S3 (microsomes and cytosolic proteins) no labeling was observed (Fig 5F). Larger peroxisomes were pelleting either in P2 or even in P1. In contrast, mitochondria were most enriched in P2 (= heavy mitochondrial fraction), but the lighter organelles were also found in P3 (light mitochondrial fraction, see above). The specific distributíon patterns of the organelle proteins in S2, P2, S3, and P3 (Fig 5F) demonstrates the good quality of our subcellular fractionation. The osteoblast marker OPN was mainly enriched in P2, but was also present in microsomes of S3 (Fig 5F). This distinct distribution pattern in comparison to peroxisomal or mitochondrial markers is due to the fact that OPN is present in all sub-compartments of the secretory pathway (see also immunofluorescence for OPN in Fig 4A). For further analyses of protein alterations during osteoblast differentiation, fractions S2 and P2 were used for the detection of OPN and mitochondrial proteins such as the complex III of the respiratory chain (ubiquinol cytochrome c oxidoreductase, subunit core 2 (UQCRC2, Fig 5G) and superoxide dismutase 2 (SOD2; Fig 5G) in comparison to fractions S3 and P3 for peroxisomal proteins (Fig 5H). Peroxisomal biogenesis and metabolic proteins, such as PEX13, PEX14, ABCD3, keto-acyl-CoA thiolase, and catalase increased during osteoblast differentiation with a peak at day 7 (Fig 5G), corroborating the results obtained by RT-PCR (Fig 5B and 5C) and immunofluorescence staining for PEX14 (Fig 4H–4K). Thereafter, all proteins except for ABCD3 declined in P3 (Fig 5H). PEX5, the cytoplasmic shuttling receptor for the import of the peroxisomal matrix proteins, exhibited a differential subcellular distribution. At day 7, the intensive PEX5 labelling of P3 (peroxisome-bound form) coincided with a strong abundance of thiolase and catalase inside peroxisomes, whereas at day 15 the reduction and shift of PEX5 mainly to the cytoplasm (S3) was accompanied by a reduced peroxisomal content of thiolase and catalase in comparison to the less altered peroxisomal membrane proteins PEX13, PEX14, and ABCD3 in P3. In contrast to the peroxisomal marker proteins, the protein levels of UQCRC2 and SOD2 in mitochondria increased from day 3 to 15 (Fig 5H).

### 5. The Peroxisome Numerical Abundance and Function were Modulated by PPAR Agonists and Antagonists

**Fig 6:**
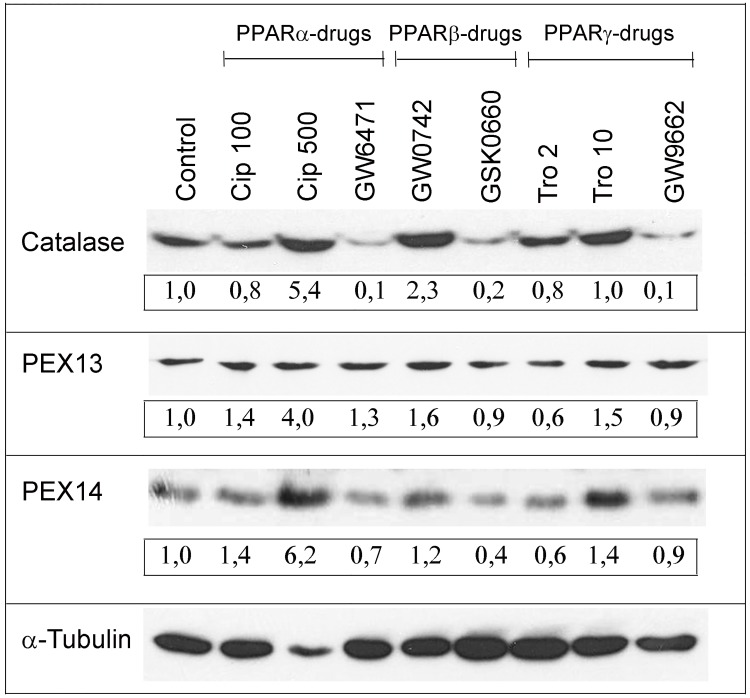
Stimulation of all three PPARs increased the peroxisome number and metabolic function in calvarial osteoblasts. Osteoblasts were exposed to agonists and antagonists of PPARɑ (Cip, GW6471), of PPARß (GW0742, GSK0660), and PPARɣ (Tro, GW9662). Cell homogenates were analyzed for the protein level of catalase, PEX14, and PEX13 using ɑ-tubulin as housekeeping protein to ensure equal protein loading on the gel. Semiquantitative analysis of the integrated optical signal intensities of the proteins related to ɑ-tubulin with controls set to 1 are shown in numbers directly below the bands.

**Fig 7:**
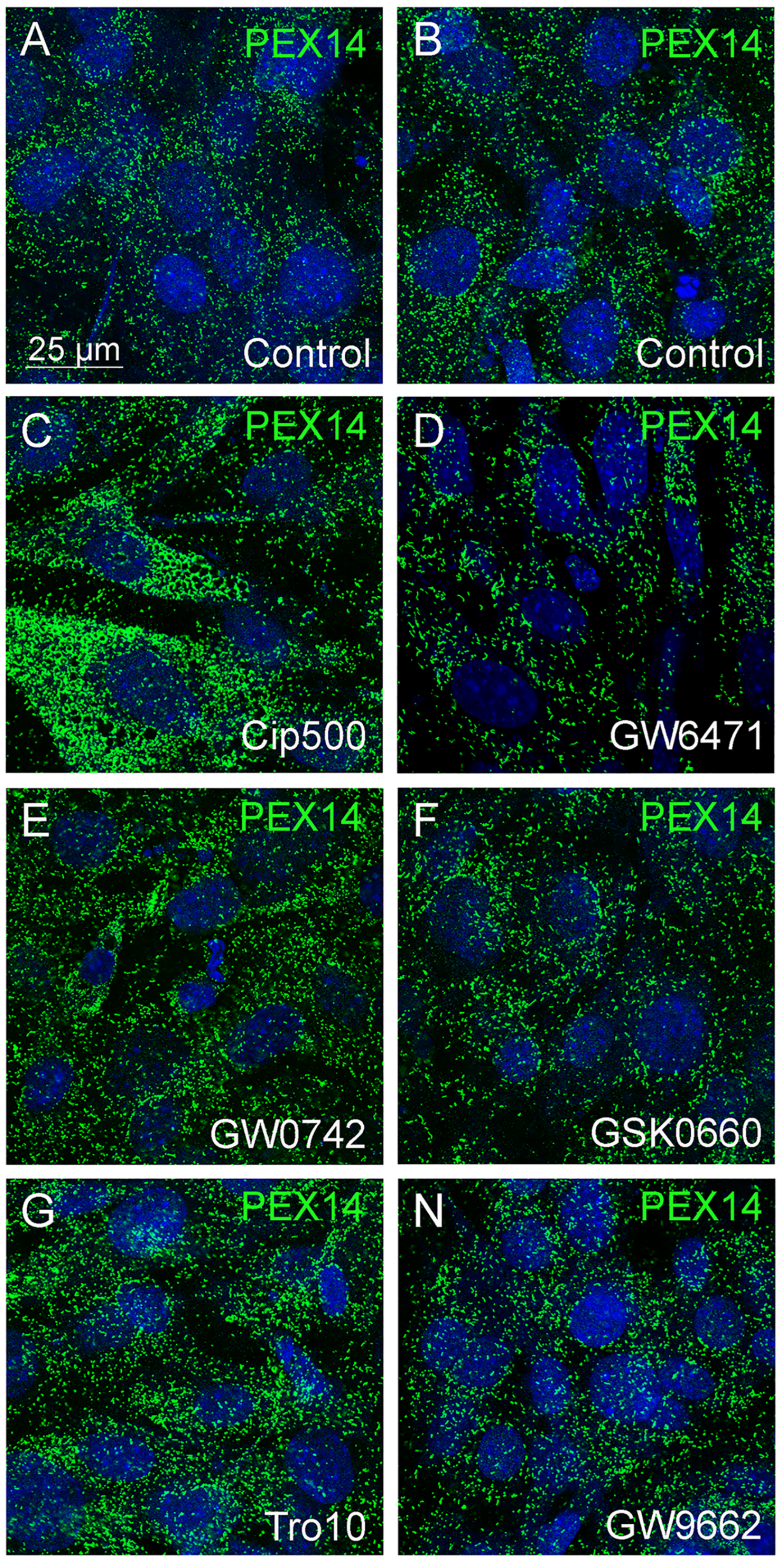
PPARɑ/ß/ɣ activation induced peroxisome proliferation in calvarial osteoblasts. (A-H) Primary osteoblasts were treated with vehicle (Vh, A, B) ciprofibrate (Cip, C), GW6471 (D), GW0742 (E), GSK0660 (F), troglitazone (Tro, G) and GW9662 (H) and were stained for PEX14. The strong immunoreactivity and homogenous distribution of PEX14 in individual peroxisomes indicates an increase in the peroxisome number (peroxisome proliferation) and not in PEX14 protein in each individual peroxisome.

It is well-known that proliferation of peroxisomes and regulation of peroxisome-related genes are induced by PPARɑ [35], but less information is available for PPARß and PPARɣ on this issue. Only for the liver, peroxisome proliferation and an increase in peroxisomal β-oxidation by treatment troglitazone (a PPARɣ agonist) and L-783483 (a dual PPARß/ɣ agonist) have been shown by DeLuca and colleagues [36]. However, the effects of both drugs were weaker in PPARɑ-knockout compared to wild-type mice [19] indicating an interaction between the three PPAR family members. Therefore, we analyzed the effects of distinct PPAR agonists and antagonists on the peroxisomal compartment in primary osteoblasts after 4 days in culture plus subsequent 6-day drug treatment. At this time point, osteoblasts contained the highest number of peroxisomes and differentiated into mature osteoblasts, but not yet into osteocytes. Treatment with the different PPAR agonists and antagonists exerted different effects on peroxisomal marker proteins with catalase exhibiting the strongest and PEX13 the weakest response. The PPARɑ agonist ciprofibrate (500 μM) strongly increased the protein levels of catalase and PEX14 in homogenates (Fig 6A), whereas the PPARɑ antagonist GW6471 decreased catalase and PEX14 at the protein level below that of controls (Fig 6A). In comparison to PPARɑ activation, a stronger increase of catalase and a weaker elevation of PEX14 have been observed for the PPARß agonist GW0742. The PPARß antagonist GSK0660 decreased both proteins below control levels. The PPARɣ agonist troglitazone (10 μM) showed similar effects as ciprofibrate (500 μM) with strongly increased catalase and PEX14 protein levels (Fig 6A). Similar to the PPARɑ and PPARß antagonists, GW9662, a PPARɣ antagonist, reduced catalase and PEX14 protein levels compared to untreated controls. To clarify whether the three PPAR agonists induce peroxisome proliferation or increase the PEX14 protein, drug-treated primary osteoblasts were stained for PEX14 revealing a higher numerical abundance of peroxisomes in cells exposed to ciprofibrate (500 μM; Fig 7C), GW0742 (Fig 7E) and troglitazone (10 μM, Fig 7F) in comparison to non-treated controls (Fig 7A and 7B). For all six PPAR-modulating drugs, changes in protein levels were in some, but not all cases paralleled by the respective mRNA levels (S1 Table) suggesting either a regulation at earlier time points or at the posttranscriptional level. In summary, activation and inhibition of all three PPARs modulated the peroxisomal compartment in differentiating osteoblasts.

### 6. The mRNA Levels for PPARɑ, PPARß and PPARɣ were Differentially Affected by PPAR-modulating Drugs

**Fig 8:**
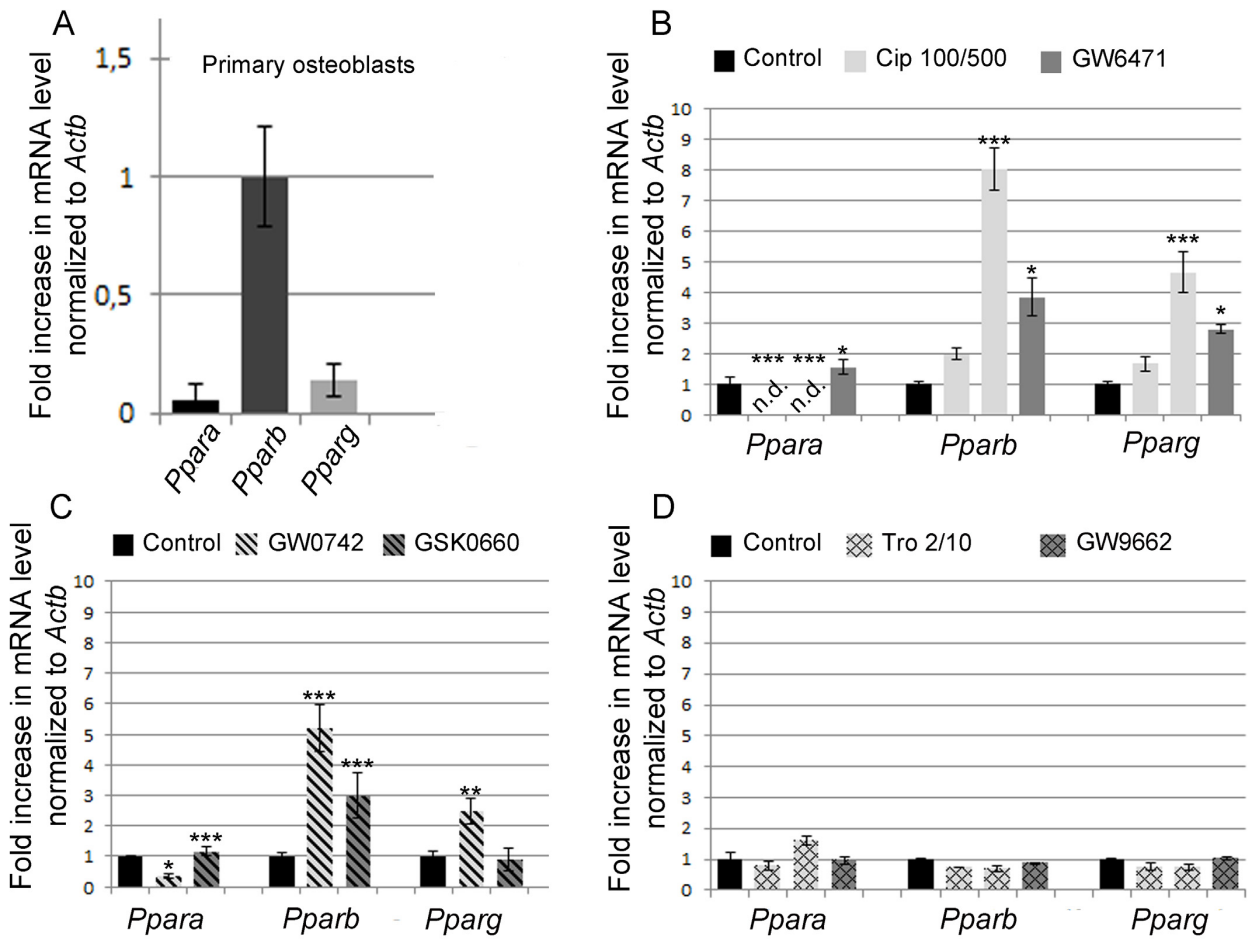
In calvarial osteoblasts, predominantly expressing PPARß, PPAR-modulating drugs affect not only the mRNA level of their own receptor, but indirectly impact the expression of the other *Ppar*s as well. (A) Comparative analysis of the mRNA levels of *Pparɑ*, *Pparß* and *Ppar* ***ɣ*** in calvarial osteoblasts after 10 days in culture. (B-D) Quantitative RT-PCR analysis of the *Ppar* mRNA levels in osteoblasts after treatment with agonists and antagonists of PPARɑ (B), PPARß (C), and PPAR**ɣ** (D). Significant differences in comparison to non-treated controls were given as *p<0.05; **p<0.01 and ***p<0.001 using ANOVA-1 followed by post-hoc Scheffé-test.

Since induction of peroxisomal proteins was found by activation of all three PPARs, we decided to analyze and to compare the mRNA levels of each family member under control and PPAR-modulating drug treatment conditions. In differentiated osteoblasts, mRNA expression values were highest for PPARß, being 200-fold higher than the one for PPARɑ and 25-fold higher than the one for PPARɣ (Fig 8A) which is in accordance with the gene expression profile data in the BioGPS data base (www.biogps.org).

Surprisingly, when primary osteoblasts were treated with the PPARɑ agonist ciprofibrate, the *Ppar*α mRNA level was down-regulated, whereas those for PPARß and PPARɣ were strongly up-regulated (Fig 8B). However, this effect was observed at 500 μM ciprofibrate (EC50 = 50 μM) which is known also to activate PPARß (EC50 >100 μM) and PPARɣ (EC50 = 500 μM). Therefore, we can´t exclude unspecific effects through PPARß and PPARɣ. The PPAR*ɑ* antagonist GW6471 increased the mRNA level of all three PPAR family members (Fig 8B). Comparable results were obtained by the activation and repression of PPARß with GW0742 and GSK0660, respectively. On the one hand, the activation with GW0742 decreased the *Pparɑ* mRNA and increased *Pparß* and *Pparɣ* mRNA levels. On the other hand, the PPARß antagonist GSK0660 elevated only the *Pparß* mRNA level (Fig 8C). Modulation of PPARɣ did not significantly affect expression levels of any of the three *Ppar* mRNAs (Fig 8D). In summary, PPAR-modulating drugs alter not only the gene expression of their own receptor, but may indirectly impact the expression of the other PPARs as well. Our results suggest that experiments studying individual PPARs in osteoblasts are complex and have to be interpreted with great care since such changes in the mRNA levels of *Ppar*s by their agonists and antagonists might in addition vary depending on the drug concentration, on the time of exposure (including positive and negative feedback mechanisms and loops) and on the ratio of the PPARs before the treatment.

### *7*. PPARß Regulates Peroxisome-related Gene Expression via the PPAR-response element (PPRE) in the Osteoblast Cell Line MC3T3-E1

**Fig 9:**
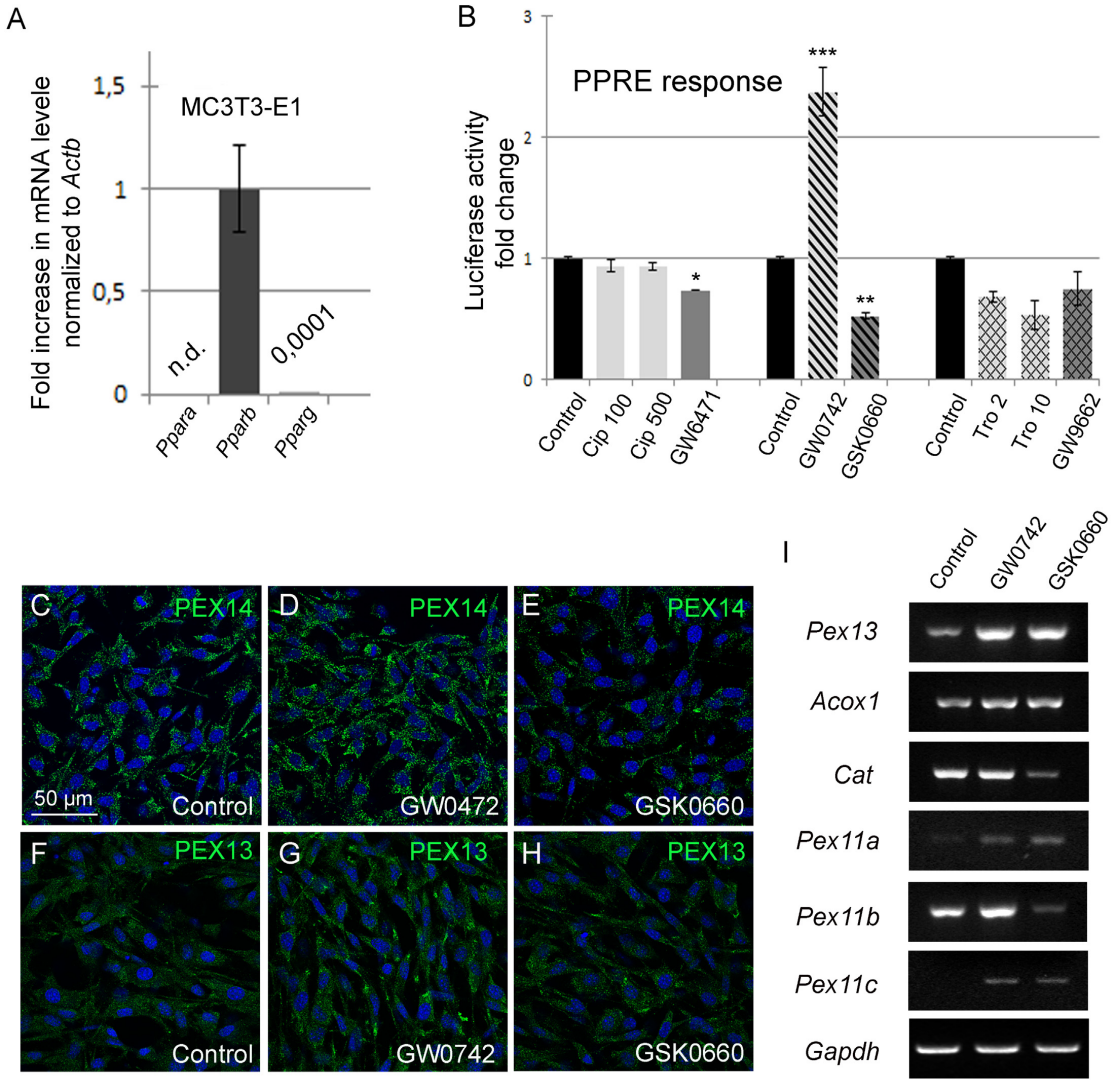
Activation of PPAR*ß* increased the peroxisome number and metabolic function in MC3T3-E1 cells. (A) Comparative analysis of the mRNA levels (qRT-PCR) of *Pparɑ*, *Pparß* and *Ppar* ***ɣ***in MC3T3-E1 cells. (B) MC3T3-E1 cells were treated with the six PPAR-modulating drugs. PPRE-activity was measured using the Dual Luciferase Reporter Gene Assay. Significant differences in comparison to untreated controls were given as *p≤0.05; **p≤0.01 and ***p≤0.001 using ANOVA-1 followed by post-hoc Scheffé-test. (C-H) Treatment of MC3T3-E1 cells with the PPARß agonist GW0742 (D, G) increased the number of peroxisomes as detected by immunofluorescence stainings for PEX14 (C-E) and PEX13 (F-H) in comparison to cells treated with vehicle (control; C, F) and the PPARß antagonist GSK0660 (E, H). G. Semiquantitative RT-PCR analysis of genes regulating peroxisome number (*Pex11)* as well as peroxisome biogenesis (*Pex13*) and metabolic function (*Cat*, *Acox1*) after treatment of MC3T3-E1 cells with GW0742.

PPAR-induced peroxisome proliferation in osteoblasts—to our knowledge—has never been reported in the literature. Since primary osteoblasts predominantly express *Pparß*, we suggest PPARß as the key player controlling peroxisome number and function. Thus, we next analyzed whether peroxisome proliferation by ligand binding to PPARß is mediated via a PPRE-dependent mechanism using a dual-luciferase plasmid-based reporter gene assay. Generally, the plasmid transfection efficiency via lipofection works better in cell lines than in primary cell cultures. For the next experiments, we therefore used MC3T3-E1 cells, a frequently used osteoblast cell line, grown for a 6 day-treatment period without passaging for the next experiments. Comparable *Pparß* mRNA levels were measured for MC3T3-E1 cells (∆ ct (*Pparß*-*Actb*) = 8,7) and primary osteoblasts (∆ ct (*Pparß*-*Actb*) = 7,3). *Pparɑ* and *Pparɣ* mRNA levels in MC3T3-E1 cells were much lower than the one for PPARß (Fig 9A; [37]) and expressed at almost undetectable levels in comparison to primary osteoblasts (Fig 8A). Among the three types of PPAR agonists, only the activation of PPARß with GW0742 significantly increased the luciferase activity (Fig 9B). Consistently, the PPARß antagonist GSK0660 repressed the PPRE activity (Fig 9B). As expected, no effects were found for drugs modulating PPARɑ activity probably due to the extremely low levels of this receptor in MC3T3-E1 cells, whereas the PPAR*ɣ* agonist troglitazone (10 μM) reduced the PPRE activity about 50% (Fig 9B). Next, we aimed to find out whether activation of PPARß would indeed induce peroxisome proliferation. For this purpose, immunofluorescence preparations to localize PEX14 (Fig 9C–9E) and PEX13 (Fig 9F–9H) were performed revealing a strong increase in peroxisome number in GW0472-treated MC3T3-E1 cells (Fig 9D and 9G) in comparison to GSK0660 (Fig 9E and 9H) and non-treated cells (Fig 9C and 9F). Next, we analyzed PPARß-induced changes in peroxisome-related gene expression. Activation of PPARß increased the mRNA levels of the β-oxidation enzyme ACOX1, the ROS degrading matrix enzyme catalase and PEX13 (Fig 9I). Vice-versa, no change or down-regulation (only catalase) of the respective gene expression levels were found after treatment with the PPARß antagonist GSK0660 (Fig 9I). Since peroxins of the PEX11 family regulate constitutive peroxisome number and peroxisome proliferation, we analyzed the gene expression of *Pex11*ß (Fig 9I). Interestingly, *Pex11ß* mRNA was highly expressed already in non-treated MC3T3-E1 cells similar to the strong staining of osteoblasts in *in situ* hybridization preparations (Fig 3). After treatment with the PPARß agonist GW0742, *Pex11ß* mRNA was strongly up-regulated and vice-versa down-regulated with the PPARß antagonist GSK0660. In contrast, *Pex11ɑ* and *Pex11ɣ* mRNAs were expressed at low levels and only slightly changed (Fig 9I) suggesting that the strong proliferation of peroxisomes after PPARß agonist treatment is mediated by PEX11ß in a PPRE-dependent manner. We confirmed the semiquantitative RT-PCR data in Fig 9I in MC3T3-E1 cells as well as in primary calvarial osteoblasts by quantitative RT-PCR analysis (Table 5).

**Table 5:** Activation of PPARß induced the expression of genes related to peroxisome proliferation and metabolic function. Primary calvarial osteoblasts and MC3T3-E1 cells were treated with the PPARß agonist GW0742 (30 μM) and the PPARß antagonist GSK0660 (150 nM) and we analyzed the mRNA levels (qRT-PCR) of the indicated genes. Significant differences between the means ± SD (n = 4) of non-treated and drug-treated cells were given as: *p≤0.05; ***p≤0.001.

*Gene*	vehicle set to1	GW0742 fold increase normalized to *Gapdh*	GSK0660 fold increase normalized to *Gapdh*
*Primary calvarial osteoblasts*			
*Pex11a*	1,00 ± 0,34	3,48 ± 0,30***	1,16 ± 0,20
*Pex11b*	1,00 ± 0,32	1,94 ± 0,40*	0,95 ± 0,47
*Pex11g*	1,00 ± 0,41	0,94 ± 0,24	0,79 ± 0,24
*Acox1*	1,00 ± 0,28	1,73 ± 0,25*	1,15 ± 0,58
*Cat*	1,00 ± 0,30	1,95 ± 0,36*	1,04 ± 0,29
*MC3T3 cells*			
*Pex11a*	1,00 ± 0,31	1,53 ± 0,44	0,87 ± 0,22
*Pex11b*	1,00 ± 0,45	2,93 ± 0,39*	0,80 ± 0,35
*Pex11g*	1,00 ± 0,56	7,81 ± 1,27***	2,69 ± 0,84*
*Acox1*	1,00 ± 0,34	3,63 ± 0,93***	1,70 ± 0,64
*Cat*	1,00 ± 0,50	1,40 ± 0,21	0,45 ± 0,17*

### 8. PPARß Accelerates Differentiation of Primary Calvarial Osteoblasts in Culture

**Fig 10:**
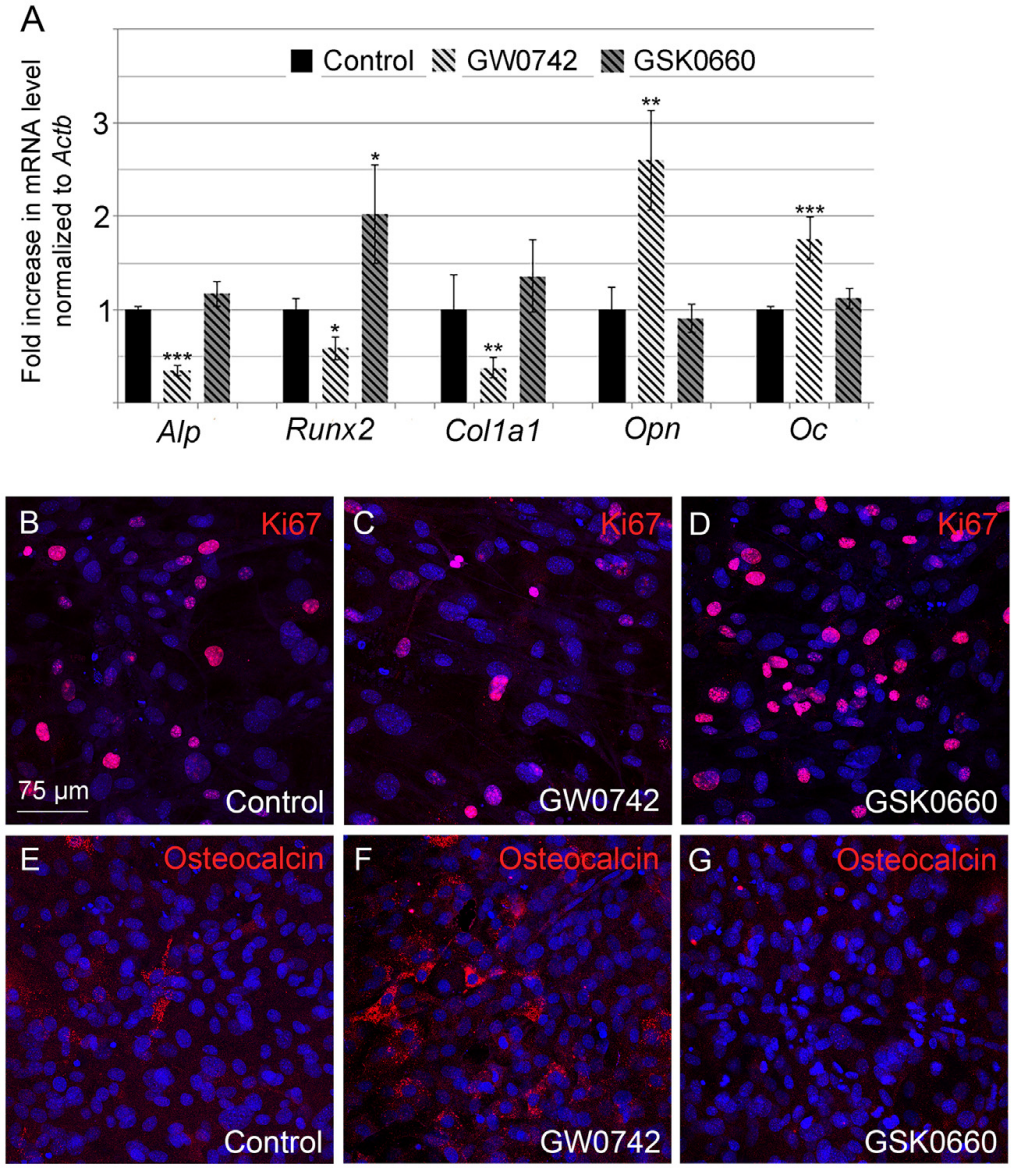
Activation of PPARß accelerated osteoblast differentiation and maturation. (A) Gene expression profile of early (*Alp*, *Col1ɑ*1), middle and late stage (*Opn*, *Oc*) markers as well as of *Runx2* which is expressed at all stages of osteoblast differentiation. (B-G) Evaluation of cell proliferation (by detecting ki67, B-D) and maturation (by detecting osteocalcin, E-G) of osteoblasts treated with the PPARß agonist GW0742 and the PPARß antagonist GSK0660.

Since we found an increase in peroxisome number on the one hand during osteoblast differentiation (Figs 4 and 5) and on the other hand by activating PPARß (Figs 6, 7 and 8), we finally investigated PPAR-induced changes in the differentiation of osteoblasts. In osteoblast cultures treated with the PPARß agonist GW0742, the middle to late stage markers OPN and osteocalcin increased at the mRNA (Fig 10A) and protein levels (Fig 10E–10H) accompanied by a decrease in the mRNA level of the early stage markers *Alp*, and *Col1*ɑ*1* (Fig 10A) in comparison to non-treated controls. The PPARß antagonist GSK0660 exerted no effect (Fig 10A and 10E–10J). Similarly, cell proliferation was lower in cultures exposed to GW0742, but remained unchanged in the presence of GSK0660 compared to non-treated controls (Fig 10B–10D).

## Discussion

In the present study, the peroxisomal compartment has been characterized in different cell types of the mouse skeleton as well as in cultured murine osteoblasts during differentiation and upon treatment with different PPAR-modulating drugs. Our findings of a link between osteoblast differentiation, peroxisome proliferation and activation of PPARß are discussed in relationship to bone and cartilage cell biology and mineralization.

### 1. Heterogeneous Numerical Abundance of Peroxisomes in Different Cell Types of the Mouse Skeleton as well as during Differentiation of Calvarial Osteoblasts in Culture

Although the importance of peroxisomes for bone development can be concluded from the severe ossification defects in patients with peroxisome biogenesis disorders and corresponding knockout mouse models [13,14,38] to date no comprehensive study exists on the distribution and function of this organelle in distinct cell types of the mouse skeleton. In 1992, Deahl et al. [39] performed enzyme-based immunohistochemistry to localize catalase, ACOX1 and keto-acyl-CoA thiolase in rat skeletal tissues, however, with unsatisfactory staining patterns. In the femora of 4 to14 week-old rats, catalase was shown to be present in articular and most prominent in epiphyseal chondrocytes and osteocytes, but was undetectable in osteoblasts, osteoprogenitor cells and bone lining cells [39]. Moreover, the diffuse staining for catalase in rat hypertrophic chondrocytes led to the assumption of an extracellular or plasma membrane-associated localization of this enzyme [39]. Also in later studies, ACOX1 and thiolase were referred to be expressed at extremely low and catalase at undetectable levels in rat vertebrae and ribs up to E19.5 [40]. However, stainings for peroxisomal proteins with metabolic functions are not ideal to label the whole population of peroxisomes in different cell types, tissues and organs and during pre- and postnatal development [31,41,42,43]. Therefore, we used PEX14 to analyze and to compare the abundance of the peroxisomal compartment independent of its metabolic function. We were indeed able to visualize this organelle in all cell types of the mouse skeleton with highest numerical abundance in mature osteoblasts, osteoclasts as well as hypertrophic chondrocytes. During differentiation from osteoblast precursor cells into mature osteoblasts, peroxisomes increased in number, whereas we observed a decrease during their further development into osteocytes within the mineralized matrix. During endochondral ossification, we detected a clear gradient with chondrocytes in the reserve zone exhibiting the lowest and those in the hypertrophic zone the highest number of this organelle. Interestingly, the high number of peroxisomes observed in hypertrophic chondrocytes was comparable to the one in osteoblasts in the ossification zone possibly implicating an important and protective role of this organelle during matrix deposition. This gradient is even more pronounced for ABCD3 and catalase showing very high levels in hypertrophic chondrocytes.

In general, peroxisomes are involved in the synthesis and degradation of a variety of bioactive lipids [1,17]. The high abundance of peroxisomes in differentiating osteoblasts and hypertrophic chondrocytes might therefore contribute to the synthesis of molecules (i) that are highly enriched in matrix vesicles initiating mineralization (e.g. lysophospholipids, phosphatidylserine) [44,45,46]) (ii) that influence the fluidity of the plasma membrane [47] thereby altering the budding of matrix vesicles and (iii) that modulate the lipid rafts of the matrix vesicles and thus the Ca^2+^-dependent annexin-binding and matrix mineralization [48,49,50,51,52,53]. Interestingly, we found the highest peroxisomal numerical abundance in the apical region of secretory ameloblasts and odontoblasts during differentiation, whereas mitochondria and other cell organelles were located more basally [54]. This is in line with the observation of retarded dentitions, malposition of teeth and enamel and dentin hypoplasia in milder forms of PBDs with prolonged survival [55]. In addition, long chain polyunsaturated fatty acids (LCPUFAs), which are metabolized in peroxisomes, are beneficial for bone turnover and thus for osteoblast differentiation (reviewed by [56,57]). Especially eicosapentanoic acid and docosahexanoic acid, which are exclusively synthesized from α-linolenic acid in peroxisomes [58], induced differentiation of mesenchymal stem cells into the osteoblast lineage by up-regulating Runx2 [59], and indirectly by reducing lipid peroxidation and protecting against the production of inflammatory cytokines [57]. Similarly, peroxisomes control the homeostasis of prostaglandins [60, 61]. Low levels of prostaglandin E2 are known to induce early osteoblast differentiation (reviewed by [57]) by increasing the activity of Runx2, BMPs [62], and the osteogenic Wnt signaling [63]. Some other lipid mediators which are in part metabolized in the peroxisomes, such as the retinoids [64] and gonadal steroids, are also known to play an important role in osteoblast differentiation and mineralization.

Altogether, peroxisomal metabolism is linked to many factors influencing intramembranous and endochondral ossification explaining our observation of an increasing number and maturation of this organelle in hypertrophic chondrocytes and in osteoblasts from the stage of a pre-osteoblast to the differentiated osteoblast.

### 2. Peroxisome Proliferation and Osteoblast Differentiation Increased upon Activation of PPARß

We have shown that osteoblast differentiation as well as activation of PPARß is accompanied by an increase in peroxisome number and an up-regulation of peroxisome-related genes such as *Pex11ɑ*, *Pex11ß*, catalase, and *Acox1* (except *Acox2*). Interestingly, peroxisome proliferation and the above mentioned genes are regulated by the binding of the ligand-activated nuclear receptor PPARɑ to its responsive element in their promoter region [65]. To date, the PPARs comprise a nuclear hormone receptor superfamily with three subtypes and 4 members, PPARɑ, PPARß/ɗ, and PPARɣ_1/2_ [66]. Induction of signaling pathways through PPARɑ varies among species and tissues which seemed to be caused by differences (i) in the PPARɑ expression level [67], (ii) in intrinsic DNA binding elements [68,69] and (iii) in the metabolic requirement of the cell. Our quantitative data showed that of the three family members, the *PPARß* mRNA level is predominantly expressed in murine osteoblasts and MC3T3-E1 cells. PPARɑ and PPARɣ mRNA levels were still detectable in primary mouse osteoblasts, and were almost absent in MC3T3-E1 cells. Therefore, easy transfectable MC3T3-E1 cells and primary calvarial osteoblasts served in our study as models to analyze signaling through PPARß with only minor interference of the other two PPARs.

Although PPARs of all three subtypes form heterodimers with RXRs and bind to a consensus PPRE [70], the other two receptors, PPARß and PPARɣ, were suggested not to work as classical peroxisome proliferators [71]. However, PPARɣ and dual PPARɣ/ß agonist were shown to increase the expression of PPARɑ target genes—such as *Acox1* and *Fabp—*independent of PPARɑ [36], suggesting an overlapping function of all three PPARs. Since the *Pex11* gene family is responsible for peroxisome proliferation and all three subtypes were up-regulated by PPARß activation in MC3T3-E1 cells, we performed a database search on mouse genome (www.sabiosciences.com/chippcrresearch) to find out whether the different *Pex11* genes contain a putative PPRE. The *Pex11ɑ* gene (inducible form) contains a PPRE for PPARɣ and PPARɑ which is known to be the signaling cascade for classical peroxisome proliferators such as the fibrates [65] even though proliferation of peroxisomes is also possible in the absence of *Pex11ɑ* [72]. The *Pex11ß* gene (constitutive form) contains a PPRE for PPARɣ, whereas the *Pex11ɣ*gene (with a yet unknown function [9]) lacks a PPRE. Interestingly, *Pex11ß* knockdown decreased PPARɣ, but not *Pparɑ* mRNA levels in Xenopus laevis oocytes [73], whereas *Pex11ß* overexpression increased the *Pparɑ* and decreased the *Ppar*ɣ mRNA levels [74]. In both cases, the mRNA levels of *Pparß* remained unchanged. Consistently, differentiated osteoblast after 10 days in culture possess a higher number of peroxisomes (Fig 4) and were found to express higher mRNA levels for PEX11ß and PPARɑ (Fig 5), constant ones for PPARß and lower ones for PPARɣ compared to undifferentiated pre-osteoblasts (Fig 5). Since not only the number of peroxisomes, but also PEX14, catalase and ACOX1 proteins were concomitantly up-regulated, we performed a database search (www.sabiosciences.com/chippcrresearch) for the transcriptional regulation of these mouse genes. This analysis revealed putative PPARɑ binding sites for *Acox1* [65] and *Cat* [75,76] genes as well as PPARɣ binding sites for the *Pex14* and *Cat* genes. Thus, PPREs were found in all “peroxisomal” genes induced by treatment with PPARß activators suggesting an overlapping activity of PPARɑ/ß/ɣ on the PPREs. Consistent with our data in Fig 9, PPREs were found in the genes for PPARɑ (PPRE for PPARɣ) and PPARß (PPRE for PPARɑ and PPARɣ), but not for PPARɣ. In agreement with our results, a tight interaction of the three PPARs, with PPARß having a central role in this PPAR trias, has been hypothesized by Aleshin et al. [77].

Since we found an increase in peroxisome number during osteoblast differentiation as well as after activation of PPARß, we finally confirmed this connection by showing an enhanced osteoblast differentiation upon activation of PPARß. Most information with regard to osteoblast differentiation is available on PPARɣ. PPARɣ is known to differentiate mesenchymal stem cells and transdifferentiate mature osteoblasts into adipocytes and to inhibit osteoblastogenesis [21,78,79]. Likewise, PPARɣ is suppressed during osteoblastogenesis [80] and differentiation (Fig 5) and the PPARɣ knockdown enhanced osteoblastogenesis [81,82]. Dual agonists for PPARɑ/PPARß [18] and the PPARɑ agonist and peroxisome proliferator fenofibrate [83] induced osteoblast differentiation. Although PPARɑ knockout mice exhibited no obvious bone phenotype and normal osteoblast differentiation [19], the PPARɑ antagonist GW6471 inhibited differentiation of periosteal cells into osteoblasts [84]. Recently, PPARß was recognized as a key regulator of bone turnover inducing osteoblast differentiation by amplification of *Wnt*-dependent and *β-catenin*-dependent pathways [20]. Consistently, PPARß knockout animals were significantly smaller at every stage of development [85]. In our study, activation of PPARß –as a novel finding—was found to increase the expression of peroxisome-related genes (e.g. *Cat*, *Acox1*, *Pex11*, and *Pex14*) as well as the numerical abundance of this organelle which coincided with osteoblast differentiation.

Bone modeling and growth during embryonic development as well as bone remodeling in adult skeleton both involve osteoblastic bone formation and osteoclastic bone resorption. In this respect, it is of interest that PPARs also play a role in osteoclasts which mainly express PPARß and PPARɣ together with a high number of peroxisomes. Since endogenous PPAR ligands are degraded in peroxisomes and PPARs regulate peroxisome number and metabolism, we suggest that a peroxisome/PPAR feedback loop is keeping the homeostasis of different lipid ligands and other factors thereby influencing bone remodeling.

In conclusion, our data showed that the peroxisomal compartment is highly abundant in hypertrophic chondrocytes, mature osteoblasts and osteoclasts. We suggest a vital role of this organelle and its metabolic function for intramembranous and endochondral ossification which is also evident from patients with peroxisomal disorders and corresponding knockout mouse models. In addition, we assume that endogenous and synthetic PPAR agonists and antagonist affect osteoblast differentiation in a complex manner which has to be taken into account for future experiments and treatment strategies for patients.

## Supporting Information

S1 Table Activation of all three PPARs increased the expression of the peroxisomal genes *Cat*, *Pex13* and *Pex14*. Primary calvarial osteoblasts were treated with the indicated drugs and were analyzed for the *Cat*, *Pex13* and *Pex14* mRNA levels by qRT-PCR. Significant differences between the means ± SD (n = 4) of non-treated versus drug-treated osteoblasts: *p≤0.05; **p≤0.01; ***p≤0.001.
(DOCX)

## Abbreviations

ACOX1/2: acyl-CoA oxidase 1/2
ALP: alkaline phosphatase
BSA: bovine serum albumin
HM: homogenization buffer
ɑ-MEM: ɑ -Minimum Essential Medium
MFP: multifunctional protein
OB: osteoblast
OC: osteocyte
OPN: osteopontin
PBD: peroxisomal biogenesis disorder
PBS: phosphate-buffered saline
PEX: peroxin
PPAR: peroxisome proliferator-activated receptor
PPRE: PPAR response element
RT-PCR: reverse transcriptase polymerase chain reaction
SD: standard deviation
SSC: standard saline citrate buffer
TBS: Tris-buffered saline
UQCRC2: ubiquinol cytochrome c oxidoreductase, subunit core 2
