# Supplementary material for: Peroxisomes in Different Skeletal Cell Types during Intramembranous and Endochondral Ossification and Their Regulation during Osteoblast Differentiation by Distinct Peroxisome Proliferator-Activated Receptors
Source: PLoS One. 2015 Dec 2;10(12):e0143439. doi: 10.1371/journal.pone.0143439 (PMC4668026; doi:10.1371/journal.pone.0143439)
Supplement: S1 Table — Primary calvarial osteoblasts were treated with the indicated drugs and were analyzed for the Cat, Pex13 and Pex14 mRNA levels by qRT-PCR. Significant differences between the means ± SD (n = 4) of non-treated versus drug-treated osteoblasts: *p≤0.05; **p≤0.01; ***p≤0.001. (DOCX) [file pone.0143439.s001.docx]

S1 Table. Activation of all three PPARs increased the expression of the peroxisomal genes *Cat*, *Pex13* and *Pex14*. Primary calvarial osteoblasts were treated with the indicated drugs and were analyzed for the *Cat*, *Pex13* and *Pex14* mRNA levels by qRT-PCR. Significant differences between the means ± SD (n=4) of non-treated versus drug-treated osteoblasts: *p≤0.05; **p≤0.01; ***p≤0.001.

|  | *Cat* mRNA level fold increase normalized to Actb | *Pex13* mRNA level fold increase normalized to Actb | *Pex14* mRNA level fold increase normalized to Actb |
| --- | --- | --- | --- |
| *Primary osteoblasts* | |  |  |
| Control set to1 | 1,00 ± 0,14 | 1,00 ± 0,33 | 1,00 ± 0,15 |
| Cip 100 | 2,44 ± 0,89** | 2,36 ± 0,61** | 2,35 ± 0,31* |
| Cip 500 | 1,96 ± 0,39* | 1,08 ± 0,12 | 1,98 ± 0,39 |
| GW6471 | 1,00 ± 0,26 | 1,05 ± 0,10 | 1,64 ± 0,26 |
| GW0742 | 1,30 ± 0,08* | 1,35 ± 0,38 | 0,76 ± 0,04 |
| GSK0660 | 1,39 ± 0,13 | 1,33 ± 0,33 | 1,66 ± 0,27 |
| Tro2 | 1,40 ± 0,19 | 1,08 ± 0,13 | 0,97 ± 0,08 |
| Tro10 | 1,83 ± 0,20 | 1,16 ± 0,10 | 1,33 ± 0,04* |
| GW9662 | 2,69 ± 0,25*** | 0,82 ± 0,07 | 0,89 ± 0,06 |
